# Performance Comparison of Droplet Digital PCR and Next‐Generation Sequencing for Circulating Tumor DNA Detection in Non‐Metastatic Rectal Cancer

**DOI:** 10.1002/cam4.70943

**Published:** 2025-05-09

**Authors:** Säde Szeto, Soili Kytölä, Erdogan Pekcan Erkan, Maarit Ahtiainen, Jukka‐Pekka Mecklin, Teijo Kuopio, Ville Sallinen, Anna Lepistö, Laura Koskenvuo, Laura Renkonen‐Sinisalo, Anu Anttonen, Kukka Heiskala, Sanni Tulokas, Siru Mäkelä, Erkki‐Ville Wirta, Tuija Tuunanen, Tapio Salminen, Ari Ristimäki, Toni T. Seppälä

**Affiliations:** ^1^ Applied Tumor Genomics Research Program, Research Program Unit, Faculty of Medicine University of Helsinki Helsinki Finland; ^2^ Department of Genetics, HUS Diagnostic Center Helsinki University Hospital Helsinki Finland; ^3^ Department of Genetics University of Helsinki Helsinki Finland; ^4^ Faculty of Medicine and Health Technology, Tampere University and Tays Cancer Centre, Tampere University Hospital Tampere Finland; ^5^ Department of Molecular Pathology Central Finland Hospital Nova, Wellbeing Services County of Central Finland Jyväskylä Finland; ^6^ Department of Education and Science Central Finland Hospital Nova, Wellbeing Services County of Central Finland Jyväskylä Finland; ^7^ Faculty of Sports and Health Sciences University of Jyväskylä Jyväskylä Finland; ^8^ Department of Biological and Environmental Science University of Jyväskylä Jyväskylä Finland; ^9^ Department of Gastroenterological Surgery Helsinki University Hospital and University of Helsinki Helsinki Finland; ^10^ Transplantation and Liver Surgery Helsinki University Hospital and University of Helsinki Helsinki Finland; ^11^ Department of Oncology HUS Comprehensive Cancer Centre and University of Helsinki Helsinki Finland; ^12^ Department of Gastroenterology and Alimentary Tract Surgery Tampere University Hospital Tampere Finland; ^13^ Department of Oncology Tampere University Hospital Tampere Finland; ^14^ Department of Pathology HUS Diagnostic Center, Helsinki University Hospital Helsinki Finland

**Keywords:** circulating tumor DNA, ctDNA, ddPCR, NGS, rectal cancer

## Abstract

**Background and Objectives:**

Circulating tumor DNA (ctDNA) can potentially identify rectal cancer patients benefiting from neoadjuvant and adjuvant therapy. This study compared droplet digital PCR (ddPCR) and next‐generation sequencing (NGS) for ctDNA detection in localized rectal cancer before and after surgery.

**Methods:**

Pre‐therapy plasma and rectal tumor samples were collected from a development group (*n* = 41) and a validation group (*n* = 26). Mutations in tumor samples were identified using NGS, and ctDNA detection was performed with both ddPCR and NGS. Recurrence was assessed 1 year after surgery in the development group.

**Results:**

In the development group, ddPCR detected ctDNA in 24/41 (58.5%) and NGS panel in 15/41 (36.6%; *p* = 0.00075) of the baseline plasma. In the validation group, 21/26 (80.8%) patients had detectable ctDNA in the pre‐therapy plasma. A positive ctDNA result was associated with higher clinical tumor stage and with lymph node positivity as detected by MRI. Postoperative ddPCR did not detect ctDNA before most recurrences.

**Conclusions:**

We demonstrated a practical oligomarker ctDNA test for localized rectal cancer suitable for clinical workflow, and that ddPCR detects ctNA from pre‐therapy plasma at a satisfactory level in advanced rectal cancers. Detecting ctDNA with ddPCR may help to assess the local severity, but the clinical utility of this approach should be evaluated in clinical trials.

**Abbreviations**
(y)pTNM: TNM staging in final pathologic examination
cfDNA: cell‐free DNA
CRC: colorectal cancer
ctDNA: circulating tumor DNA
cTNM: clinical TNM staging
ddPCR: droplet digital PCR
DNA: deoxyribonucleic acid
EMVI: extramural vascular invasion
mrEMVI+: extramural vascular invasion in rectal MRI
MRI: magnetic resonance imaging
NGS: next‐generation sequencing
SYNCOPE: SYstemic Neoadjuvant and adjuvant COntrol by PrEcision medicine in rectal cancer
TME: total mesorectal excision
TNM: tumor‐node‐metastasis
VAF: variant allele frequency

## 1 Introduction

Colorectal cancer (CRC) causes the second most cancer‐related deaths worldwide [1] and rectal cancer covers roughly a third of all CRCs [2]. Neoadjuvant (chemo)radiotherapy followed by total mesorectal excision (TME) is mostly recommended for patients with stage II–III rectal cancer [3, 4]. With modern therapies, local recurrences have become reasonably rare [5]. Still, roughly a third of rectal cancers evolve distant metastases [6], partially causing a reduced 5‐year survival rate for all CRC patients (60%) [7]. Adjuvant chemotherapy is used to reduce recurrences in “high‐risk” stage II and stage III rectal cancer [3, 4]. Solid evidence of survival benefit from adjuvant therapy in rectal cancer is lacking [8, 9, 10, 11], possibly as a consequence of the incapability to classify patients to high recurrence risk patients as meticulously as needed. Several high‐risk factors besides the high tumor‐node‐metastasis (TNM) stage have been discovered, including extramural vascular invasion (EMVI) and lymphatic invasion in magnetic resonance imaging (MRI), low tumor differentiation grade, ≤ 1 mm circumferential margin, high or intermediate budding, and poor quality of TME specimen [12, 13, 14].

Circulating cell‐free DNA (cfDNA) is fragmented DNA circulating in the blood released by physiological and pathological mechanisms. In patients with cancer, a fraction (0.01%–< 10%) of cfDNA originates from malignant cells, called circulating tumor DNA (ctDNA) [15, 16, 17]. Detecting somatic alterations originating from primary tumors using peripheral blood (“liquid biopsy”) enables minimally invasive monitoring of cancer [18].

Droplet digital PCR (ddPCR) is a specific, mutation‐driven ultrasensitive assay for ctDNA detection that measures the absolute number of targeted DNA mutations, and for which individual detection probes are easy to design. Panel sequencing with the next‐generation sequencing (NGS) technique can be used to detect multiple somatic alterations in a single assay, but it is not as sensitive as ddPCR [17, 19]. Compared to NGS, ddPCR allows the operational costs of ctDNA detection with ddPCR are 5–8.5‐fold lower [20]. While ddPCR is generally a low‐cost assay, its overall expenses depend on the frequency of the targeted mutation, as custom probes for rare mutations may not be practical due to high costs.

The presence of ctDNA in early‐stage CRC is reasonably high [21]. Utilizing ctDNA preoperatively to assess the molecular tumor burden and postoperatively for detecting residual disease for recurrence risk assessment are the most potential clinical applications of ctDNA [22, 23]. Patients with ctDNA detected after curatively intended therapy have a high recurrence risk in stage II–III CRCs (up to 80%–100%) [24, 25, 26, 27, 28]. In rectal cancer, the clinical utility of ctDNA has not been validated yet, but there are few studies suggesting that serial ctDNA detection could be implemented in clinical practice [29, 30]. Still, research on ctDNA detection among rectal cancers with multiple different assays side by side is lacking.

Here, we compared the performance of tumor‐informed ctDNA assay (ddPCR) to tumor‐uninformed assay (NGS) in non‐metastatic rectal cancer. The study tested the clinical utility of in‐house tumor‐informed ctDNA assay to precede a clinical trial aiming at implementing a method to clinical decision‐making. We assessed the presence of ctDNA in different stages of rectal cancer before any therapy and compared the detection rate with different variables, aiming to determine the significance of pre‐therapy ctDNA detection for postoperative recurrence risk.

## 2 Materials and Methods

### 2.1 Study Design and Patients

This prospective observational study consisted of a longitudinal cohort of development‐phase patients and a distinct validation set of the first 26 patients enrolled in a clinical trial (NCT04842006).

The development cohort had 46 non‐metastatic sporadic rectal cancers operated at Helsinki University Hospital, Helsinki, Finland, during 2019–2020, planned for curative‐intended surgery. Primary tumor specimens, baseline plasma samples, and for 25 patients, follow‐up plasma samples were collected. All specimens were prospectively collected and associated with clinical data. The patients were recruited as control patients in a study protocol [31], of which we included 46 patients with various rectal cancer stages and risk factors.

The validation cohort included tumor specimens and baseline plasma samples of 26 first patients with non‐metastatic rectal cancer randomized to a clinical trial “SYstemic Neoadjuvant and adjuvant COntrol by PrEcision medicine in rectal cancer (SYNCOPE)”. The patients underwent neoadjuvant therapy based on the study arm they were randomized. All patients are described in more detail in the Appendix S1

### 2.2 Plasma and Tumor Tissue Collection

All baseline plasma samples were collected on the day of the first clinical visit to the tertiary center for rectal cancer before any neoadjuvant therapy was given. Follow‐up plasma samples were collected 12 months after surgery during an outpatient visit, following any adjuvant therapy. From each patient, 3 × 9 mL of blood was collected into Streck Cell Free DNA BCT (Streck, Omaha, NE, US) vacuum tubes.

Tissue specimens for tumor DNA in the development cohort were collected from the surgical resection specimen, following any neoadjuvant therapy. For the validation cohort, tumor DNA was isolated from pre‐therapy biopsies.

### 2.3 Primary Tumor DNA Panel Sequencing

To detect somatic alterations present in the primary tumor specimens, an Ion AmpliSeqTM Cancer Hotspot Panel v2 (HS1) sequencing by ThermoFisher was performed. IonAmpliseq Library Kit 2.0 with the Library Equalizer was used for library preparation. The HS1 panel has wide coverage, especially *KRAS*, *BRAF*, *APC*, and *EGFR* genes covering > 2800 COSMIC variants from 50 oncogene and tumor suppressor gene hotspot areas, with theoretical coverage of 99% in rectal patients [32, 33, 34, 35, 36]. The detection rate ranges from 98% to 5% variant allele frequency (VAF) in somatic alterations, and it has on average 2000× coverage in sequencing with 154 bp depth amplicon length. The description of tumor DNA preparation and isolation in detail is described in Supplementary Methods: Appendix S1.

### 2.4 ctDNA Detection With ddPCR and NGS

The preanalytical preparation of plasma cfDNA is described in the Supplementary Methods: Appendix S1. To detect ctDNA, two different methods were compared in the development group: ddPCR variant‐specific calling and NGS‐based panel sequencing.

ddPCR assays were performed using one to two predesigned probes, based on the mutations with the highest variant allele frequencies that occurred in the matched primary tumor NGS. Two mutations were tested with ddPCR, if feasible. ddPCR detects somatic alterations at low frequencies (VAF 0.01%) by dividing 2‐9 μL extracted DNA into 20,000 droplets and calculating the absolute quantity of targeted cfDNA based on PCR‐positive and PCR‐negative droplets.

We performed the same panel sequencing as for primary tumors (HS1 panel), optimized for ctDNA. Based on the ddPCR results, the geneticist lowered the variant calling threshold for the NGS panel sequencing. The threshold for detection of somatic alteration was 0.01% VAF (Supplementary Methods: Appendix S1).

All ctDNA analyses were performed by an experienced hospital geneticist (SK). Results were allocated to two different groups: ctDNA‐positive if any detectable ctDNA was present, and ctDNA‐negative if ctDNA was not present. Even one oncogenic mutation found in plasma was deemed ctDNA positivity.

### 2.5 Statistical Analyses

Differences in clinicopathological features between ctDNA‐positive and ctDNA‐negative patients across the follow‐up were assessed using two‐tailed Fisher's exact test for categorical variables. The Shapiro–Wilk test was performed to test whether continuous variables came from a normal distribution. Mann–Whitney (rank‐sum) test was used to compare the differences between two groups for non‐normally distributed continuous variables and ctDNA‐positive and ctDNA‐negative patients, while the *T*‐test was used for normally distributed continuous variables. The Kruskal–Wallis test was used to determine the difference between cancer stages and continuous variables. Patients' ctDNA status was defined as a combined result from both ddPCR‐ and NGS‐panel analysis for statistical analyses in the development cohort. Sensitivity or specificity was not reported as all patients were diagnosed with cancer.

Survival times were analyzed using the Kaplan–Meier algorithm. The follow‐up was defined as the time from surgery to the latest date when the medical record was reviewed for the study (9/2023). The clinical follow‐up time was from surgery to the latest colonoscopy or CT scan. The recurrence time was defined as from surgery to the first documented suspect mass on a CT scan.

All analyses were performed with R studio version 2022.07.01 (R Project for Statistical computing) with an entirely self‐produced software. A *p*‐value < 0.05 was interpreted as a statistically significant result.

## 3 Results

### 3.1 Clinicopathological Characteristics

**FIGURE 1:**
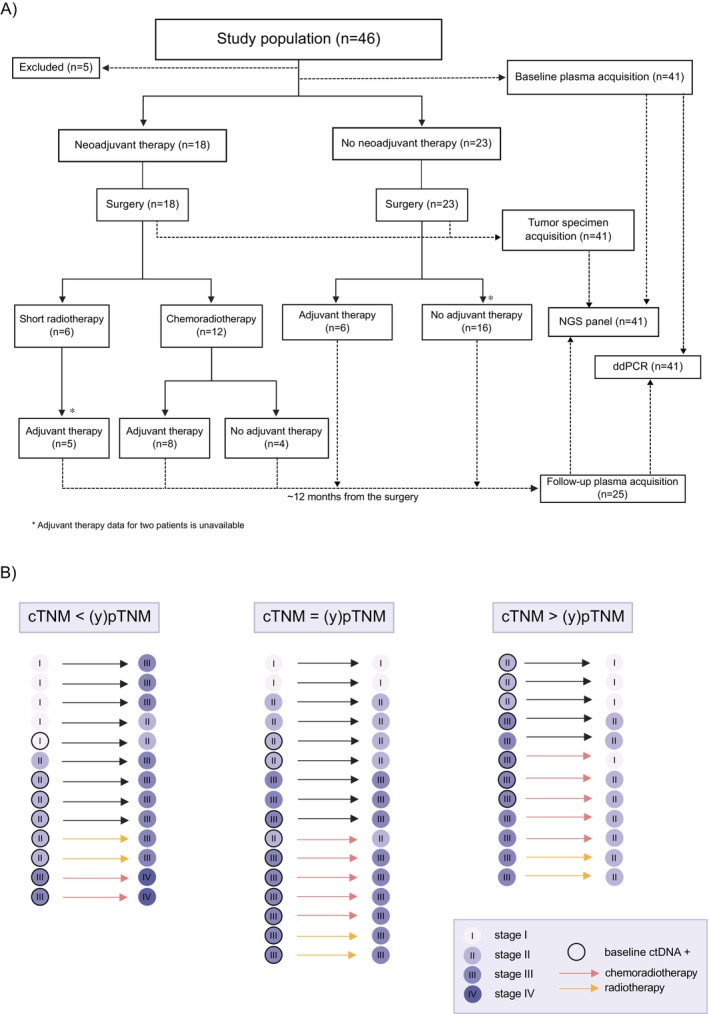
(A) A flowchart of the development cohort, patient enrollment, and study design. NGS, next‐generation sequencing; ddPCR, droplet digital PCR; short radiotherapy, short‐course radiotherapy 5 × 5 Gy; chemoradiotherapy; 50.4/1.8 Gy radiotherapy with capecitabine radiosensitizer. (B) Illustration of the discrepancy between cTNM‐status and (y)pTNM‐status with respect to baseline ctDNA status and neoadjuvant therapy. Sixteen patients had consistent staging, while 13 patients exhibited upstaging and 12 downstaging. cTNM‐status, TNM‐stage in the pre‐therapy rectal MRI. (y)pTNM‐stage, TNM‐stage in the final pathological examination. cTNM‐status, clinical TNM‐status; (y)pTNM‐status, TNM‐status in the final pathological examination.

Patient enrolment, sample collection, and multidisciplinary treatment of rectal cancer are illustrated in Figure 1A. A total of 46 patients' samples were analyzed in the development group. Five patients were subsequently excluded from the final tumor‐informed analysis as no mutations were detected from the primary tumor using the panel utilized in this study. A total of 41 patients' primary tumor and baseline plasma samples and follow‐up plasma samples from 25 patients were analyzed successfully in the development study cohort.

Demographic and clinicopathological characteristics grouped by serial ctDNA status are presented in Tables 1 and 2. The median age was 71 years, 65.9% (27/41) were male, and by initial clinical TNM staging (cTNM), 17.1% (7/41) were stage cI tumors, 34.1% (14/41) cII‐, and 48.8% (20/41) cIII‐stage tumors. Notably, 34.1% (14/41) of the tumors had EMVI on MRI (mrEMVI+).

**TABLE 1:** Clinicopathological characteristics of the development cohort with baseline ctDNA status when detected with either method utilized.

Variable	Baseline ctDNA (*n* = 41)			All patients (*n* = 41)
	Positive (*n* = 25)	Negative (*n* = 16)	*p*	
Age, years				
Median	70	74	0.34	71
Range	45–86	53–85		45–86
Sex, *n* (%)				
Male	16 (64.0)	11 (68.8)	1.00	27 (65.9)
Female	9 (36.0)	5 (31.3)		14 (34.1)
cfDNA concentration, (ng/mL)^a^				
Median	398	436	0.90	428
Range	175–955	208–1150		175–1150
Baseline CEA‐concentration, *n* (%)^a^				
Positive	8 (34.8)	2 (13.3)	0.26	10 (26.3)
Negative	15 (65.2)	13 (86.7)		28 (73.7)
Distance from anal verge, *n* (%)				
0–5 cm	4 (16.0)	2 (12.5)		6 (14.6)
5–10 cm	20 (80.0)	13 (81.3)	0.58	33 (80.5)
> 10 cm	1 (4.0)	1 (6.3)		2 (4.9)
Clinical T stage, *n* (%)				
cT1‐2	2 (8.0)	7 (43.8)	0.017	9 (22.0)
cT3‐4	23 (92.0)	9 (56.3)		32 (78.0)
Clinical N stage, *n* (%)				
cN0	12 (48.0)	9 (56.3)	0.75	21 (51.2)
cN1‐2	13 (52.0)	7 (43.8)		20 (48.8)
Clinical TNM stage, *n* (%)				
I	1 (4.0)	6 (37.5)	0.0094	7 (17.1)
II‐III	24 (96.0)	10 (62.5)		34 (82.9)
Extramural vascular invasion in MRI, *n* (%)				
Positive	10 (40.0)	4 (25.0)	0.50	14 (34.1)
Negative	15 (60.0)	12 (75.0)		27 (65.9)
Neoadjuvant therapy, *n* (%)				
Yes	14 (56.0)	4 (25.0)	0.063	18 (43.9)
No	11 (44.0)	12 (75.0)		23 (56.1)
Radiotherapy	4 (28.6)	2 (50.0)	1.00	6 (14.6)
Chemoradiotherapy	10 (71.4)	2 (50.0)	0.084	12 (29.3)
Tumor regression grade, *n* (%)^a^				
Complete‐good	6 (46.2)	2 (50.0)	1.00	8 (47.1)
Moderate‐poor	7 (53.8)	2 (50.0)		9 (52.9)
Pathologic circumferential resection margin, *n* (%)				
Positive	1 (4.0)	1 (6.3)	1.00	2 (4.9)
Negative	24 (96.0)	15 (93.8)		39 (95.1)
Differentiation, *n* (%)^a^				
Poor	1 (4.2)	0 (0.0)	1.00	1 (2.5)
High‐Moderate	23 (95.8)	16 (100.0)		39 (97.5)
Pathological T stage, *n* (%)				
(y)pT1‐2	6 (24.0)	5 (31.3)	0.72	11 (26.8)
(y)pT3‐4	19 (76.0)	11 (68.8)		30 (73.2)
Pathological *N* stage, *n* (%)				
(y)pN0	11 (44.0)	10 (62.5)	0.34	21 (51.2)
(y)pN1‐2	14 (56.0)	6 (37.5)		20 (48.8)
Pathological TNM stage, *n* (%)				
(y)pI‐II	11 (44.0)	10 (62.5)	0.34	21 (51.2)
(y)pIII‐IV	14 (56.0)	6 (37.5)		20 (48.8)
Lymph node yield, *n* (%)^a^				
< 12	7 (28.0)	1 (6.7)	0.22	8 (20.0)
≥ 12	18 (72.0)	14 (93.3)		32 (80.0)
Pathological extramural vascular invasion, *n* (%)				
Positive	11 (44.0)	7 (43.8)	1.00	18 (43.9)
Negative	14 (56.0)	9 (56.3)		23 (56.1)
Tumor cellularity, (%)^a^				
Mean	31	24	0.32	28
Range	5–70	5–60		5–70

*Note:* Tumor regression grade (mrTRG) was defined according to MRI after neoadjuvant therapies. 5‐step classification was used. Pathologic circumferential resection margin (ypCRM) was considered positive if ypCRM ≤ 1 mm and negative if > 1 mm. Carcinoembryonic antigen (CEA) was defined as positive if CEA concentration was > 5.0 μg/L, and negative if CEA ≤ 5.0 μg/L.

Abbreviations: 1, complete response; 2, good response; 3, moderate response; 4, poor response; 5, no response.

^a^ Clinicopathological characteristics unavailable; tumor differentiation (*n* = 1), lymph node yield (*n* = 1), CEA‐concentration (*n*=3), mrTRG (*n* = 1, *n* = 23 abstained neoadjuvant therapies), and tumor cellularity (*n* = 7).

**TABLE 2:** Follow‐up ctDNA status and clinical variables in development group.

Variable	Follow‐up ctDNA (*n* = 25)			All patients (*n* = 41)
	Positive (*n* = 4)	Negative (*n* = 21)	*p*	
cfDNA concentration, (ng/mL)^a^				
Median	656	507	0.53	594
Range	312–826	213–1430		213–1430
Adjuvant chemotherapy, *n* (%)^a^				
Yes	2 (50.0)	9 (42.9)	1.00	19 (48.7)
No	2 (50.0)	12 (57.1)		20 (51.3)
Metastasis location, *n* (%)				
Lungs	0 (0.0)	7 (63.6)	0.29	7 (43.8)
Liver	1 (100.0)	3 (27.3)	0.53	6 (37.5)
Distant lymph nodes	0 (0.0)	0 (0.0)	1.00	1 (6.3)
Brain	0 (0.0)	1 (9.1)	1.00	2 (12.5)
Metastasis at any site, *n* (%)				
Yes	1 (25.0)	8 (38.1)	1.00	12 (29.3)
No	3 (75.0)	13 (61.9)		29 (70.7)
Recurrence at any site, *n* (%)				
Yes	1 (25.0)	7 (33.3)	1.00	10 (24.4)
No	3 (75.0)	14 (66.7)		31 (75.6)

Abbreviations: cfDNA, cell‐free DNA; ctDNA, circulating tumor DNA.

^a^ Clinical characteristics unavailable: adjuvant therapy (*n* = 2) and follow‐up cfDNA concentration (*n* = 6).

The mean baseline plasma cfDNA concentration was 476 ng/mL. Stage cII–III tumors had higher cfDNA concentrations compared to stage cI tumors (*p* = 0.046); otherwise, no associations with clinicopathological features and cfDNA concentrations were detected (Figure S1). Less than half (43.9%, 18/41) of the patients underwent neoadjuvant therapy. In the final pathological examination, TNM‐stages ((y)pTNM) of patients included six (14.6%) (y)pI‐, 15 (36.6%) (y)pII‐, 18 (43.9%) (y)pIII‐, and two (4.9%) (y)pIV‐stage cancers. The mean cancer cellularity was 27.9% (range: 5%–70%, *n* = 34) and it was correlated with tumor tissue VAF (*R* = 0.41, *p* = 0.00015, Figure S2A). Disease‐free patients had higher VAF's in the primary tumors (*p* = 0.011, Figure S2B). Figure 1B represents cTNM‐stages, neoadjuvant regimen, and (y)pTNM‐stages with baseline ctDNA status. Of all patients, 19 (48.7%) were referred to adjuvant chemotherapy.

### 3.2 Primary Tumor Sequencing

We identified 101 hotspot gene mutations, of which 74 were unique somatic variants from 46 tumor tissue specimens (Table S1). Ten different genes were mutated with a median of 2 (range 0–5) variants called in each primary tumor (Figure S2C). No mutations were detected in five tumors (10.9%) with the 50‐gene panel used. The most frequently mutated genes were generally known driver genes *TP53* (33.7%, 34/101), *APC* (*30.7*%, 31/101), and *KRAS* (14.9%, 15/101).

### 3.3 ctDNA Detection by NGS and ddPCR in Baseline Plasma Samples

Plasma ctDNA was detected in 61.0% (25/41) of the baseline samples by either NGS or ddPCR, and only 14 (34.1%) baseline samples were ctDNA positive with both methods. NGS detected ctDNA in 36.6% (15/41), whereas tumor‐informed ddPCR with 1–2 variant markers detected ctDNA in 58.5% (24/41) of baseline plasma samples. The ctDNA detection rate for ddPCR was statistically significantly superior compared to the NGS‐based panel (*p* = 0.00075, Fishers exact test, Table 3).

**TABLE 3:** A contingency table of baseline ctDNA status detected with ddPCR and/or NGS panel.

	ddPCR positive	ddPCR negative
NGS positive	14	1
NGS negative	10	16

*Note:* A statistically significant difference between the detection rate of ctDNA between NGS panel and ddPCR was observed (*p* = 0.00075, Fisher's Exact test).

Abbreviations: ddPCR, droplet digital PCR; NGS, next‐generation sequencing.

In total, 23 patients' tumor‐informed ddPCR relied only on one variant target marker. For seven patients, we found only one mutation in the primary tumor to target with ddPCR. For sixteen patients, although more than one variant was detected in the primary tumor, only one target was used for ddPCR. Nine out of 23 of these patients were baseline ctDNA negative with ddPCR, of which five had more than one variant detected from the primary tumor. All lacked detectable ctDNA based on NGS panel sequencing, as well. When only one variant was targeted, 60.9% (14/23) of the patients were ctDNA positive, whereas 55.6% (10/18) were positive when two variants were targeted. The number of mutations detected with NGS was 0–4 and 0–2 by ddPCR. In terms of baseline ctDNA composition, mainly mutations in *TP53* (42.5%, 17/40), *APC* (20.0%, 8/40), and *KRAS* (17.5%, 7/40) (Figure S2D).

### 3.4 Associations Between ctDNA Positivity and Clinical Characteristics

**FIGURE 2:**
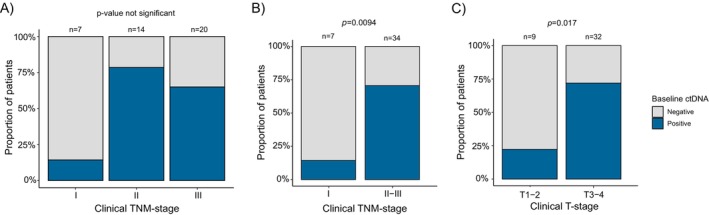
The proportion of baseline ctDNA‐positive and ‐negative patients according to the initial clinical TNM‐stage (A, B) and cT‐status (C) when both methods were utilized. cT‐status, T‐stage in the initial rectal MRI; ctDNA, circulating tumor DNA. The *p*‐values are calculated with two‐tailed Fisher's exact test.

Baseline ctDNA‐positivity was more frequently seen in locally advanced cancers, as ctDNA detection rates were 14.3% (1/7), 78.6% (11/14), and 65.0% (13/20) for cI, cII, and cIII TNM stage tumors in the baseline plasma sample (Figure 2A). A statistically significant difference between cI‐ and cII–III stages with respect to baseline ctDNA positivity was seen, as 14.3% (1/7) of cI and 70.6% (24/34) of cII–III cancers (*p* = 0.0094, Fishers exact test) had ctDNA in the baseline plasma sample (Figure 2B).

A difference in baseline ctDNA status between cT1–2 cancers and cT3‐4 cancers was evident, as 22.2% (2/9) of cT1‐2 cancers and 71.9% (23/32) of cT3‐4 cancers were baseline ctDNA positive (*p* = 0.017, Fishers exact test) (Figure 2C). However, only 65.0% (13/20) of patients with cN1‐2 tumors and 57.1% (12/21) of patients without lymph node invasion in MRI (cN0) had detectable ctDNA in baseline plasma (*p* = 0.75). Most (71.4%, 10/14) of the mrEMVI+ patients were baseline ctDNA positive, and of all patients with mrEMVI+ and cN1‐2, 75% (9/12) demonstrated detectable ctDNA at baseline.

Baseline ctDNA detection rates were 66.7% (4/6), 46.7% (7/15), 66.7% (12/18), and 100% (2/2) for I, II, III, and IV (y)pTNM‐stage tumors, respectively. Patients with pathology‐assessed vascular invasion of the primary tumor had baseline ctDNA positive plasma in 61.1% (11/18). A difference in primary tumors VAF's was not detected between baseline ctDNA positive and negative patients (Figure S2E).

Most patients with cN1‐2 (81.8%; 9/11) and (y)pN1‐2 tumors were baseline ctDNA positive. Four out of nine patients with (y)pN0‐tumors but originally cN1‐2 were baseline ctDNA positive, of which seven received neoadjuvant therapy. Roughly half (55.6%; 5/9) of patients with cN0 and (y)pN1‐2 tumors were baseline ctDNA positive, of which only two received neoadjuvant therapy.

### 3.5 Longitudinal ctDNA Analysis at Clinical Follow‐Up

The follow‐up plasma samples were collected a mean of 12 (range: 7–19 months) months after surgical resection, from which ctDNA was detected in 4/25 (16.0%) with both methods utilized. All patients with ctDNA‐positive follow‐up plasma were also ctDNA‐positive at baseline. None to three mutations were detected by NGS and none to two by ddPCR.

One out of four of the patients with ctDNA positive follow‐up plasma established a recurrence during surveillance, and three patients remained recurrent‐free during mean 33‐month surveillance from operation to the latest clinical follow‐up. Disease‐free patients with follow‐up samples are presented in detail in Figure S3. Nine out of 25 patients with follow‐up samples developed a metastatic disease during the follow‐up. Clinicopathological features with follow‐up ctDNA status are presented in Table 2.

### 3.6 Association of ctDNA With Recurrence and Survival

**FIGURE 3:**
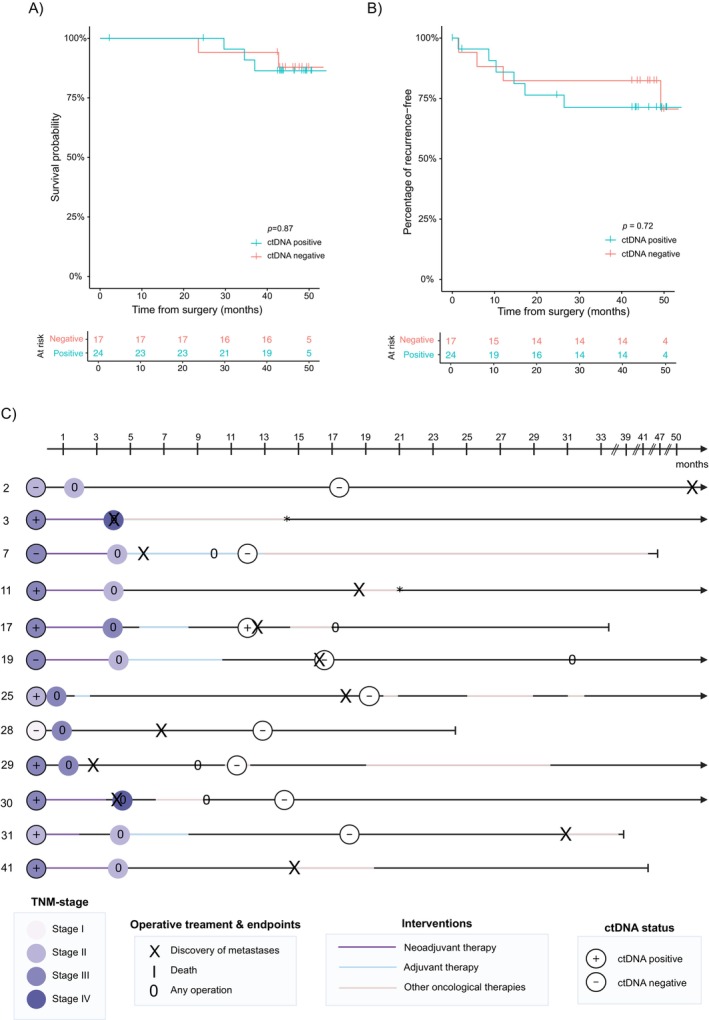
A Kaplan–Meier estimate of cancer‐related death (A) and recurrence‐free survival (B) based on baseline ctDNA status by using ddPCR alone. (C) Longitudinal ctDNA and clinical follow‐up with a swim track plot of patients with recurrence during the follow‐up. *The illustration also includes two patients diagnosed with metastatic disease before or at the time of surgery (#3 and #30). Information on the length of oncological therapies was unavailable for two patients (#3 and #11). ctDNA, circulating tumor DNA; ddPCR, droplet digital PCR. The *p*‐values are determined by the Log‐rank test.

The patients were followed up for a mean of 45 months (range: 2–54) after surgery, and the latest colonoscopy or CT scan was on average at 25 months after surgery. Eight patients (19.5%) succumbed, of which five were rectal cancer‐related deaths. A Kaplan–Meier curve of cancer‐specific survival grouped by baseline ctDNA status is presented in Figure 3A. For those who died, the mean survival after operation was 30 months (range: 2–50 months).

Ten patients out of 41 patients (24.4%) had a recurrence during follow‐up. The recurrence‐free survival is presented as a Kaplan Meier estimate assembled with baseline ctDNA status (Figure 3B). The mean time to recurrence was 15 months (range: 1–49 months) after the operation. Schematic illustration (swim track plot) of patients with recurrence and patients with detected metastases after diagnosis are presented in Figure 3C. All recurrences were distant metastases, of which seven patients' metastases were located in the lungs, four in the liver, two in the brain, and one in distant lymph nodes. In addition to these 10 patients with recurrence, two patients (#3, #30) were diagnosed with liver metastases before or during the operation. All patients who relapsed had *TP53* mutation detected in the primary tumor.

Two‐thirds (8/12) of patients with metastatic disease had cIII‐stage cancer, and 8/12 were ctDNA positive for baseline plasma. Eight out of 10 patients who had a true recurrence during the follow‐up had a follow‐up sample available. However, only one patient was ctDNA positive at 12 months, 1 month prior to the clinical diagnosis of the metastatic disease (#17).

Metastatic diseases occurred in cIII‐stage cancer (40.0%; 8/20) and final pathology stage III–IV cancers (30.0%; 6/20), but also in 19.0% (4/21) of cI‐II cancers and 28.6% (6/21) of final pathology stage I–II cancers. Only 40.0% (4/10) of the patients who had recurrence had received adjuvant chemotherapy. All individual recurrent cases have been described in the Appendix S1 discussing the ctDNA findings.

### 3.7 Performance of Tumor‐Informed ctDNA Detection in Baseline Plasma Samples in the Validation Cohort

**FIGURE 4:**
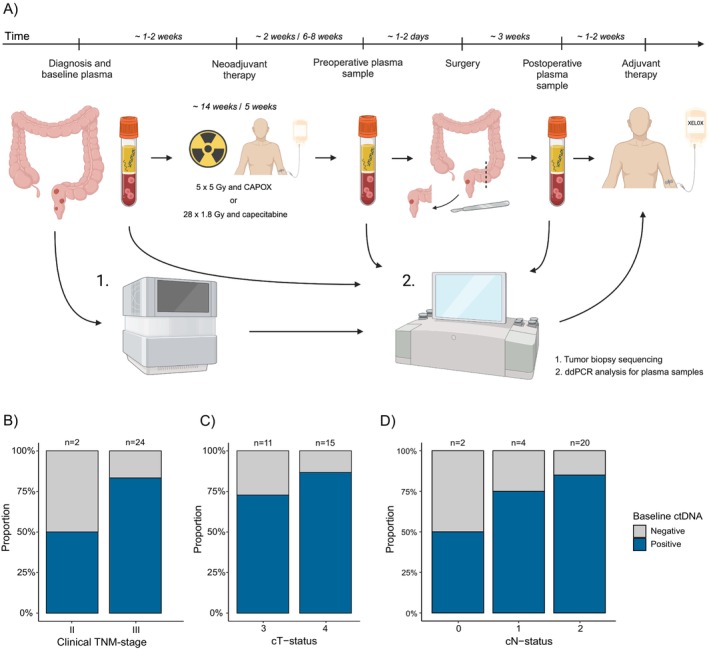
The validation cohort study design and results. (A) An illustration of the SYNCOPE trial. Baseline ctDNA status stratified by initial clinical TNM‐stages (B), cT‐status (C), and cN‐status (D). CAPOX, capecitabine‐oxaliplatin combination chemotherapy; cN‐status, N‐stage in the initial rectal MRI; cTNM‐status, clinical TNM‐status; cT‐status, T‐stage in the initial rectal MRI; ddPCR, droplet digital PCR; SYNCOPE, SYstemic Neoadjuvant and adjuvant COntrol by PrEcision medicine in rectal cancer; XELOX, Oxaliplatin and capecitabine.

The purpose of the development cohort of 46 patients was to test and prepare the ctDNA logistics for practical use in an ongoing clinical trial. As a validation cohort for tumor‐informed rectal cancer ctDNA assay with ddPCR, we utilized preliminary data from the ongoing SYNCOPE trial (NCT04842006) that is a multicenter study at Helsinki University Hospital and Tampere University Hospital launched in 2022. Patients with locally advanced rectal cancer with mrEMVI+ enrolled to this trial contributed plasma before neoadjuvant therapy. The purpose of the trial is to introduce tumor‐informed serial ctDNA measurements in multimodal therapy and multidisciplinary decision‐making for high metastasis risk rectal cancer (Figure 4A).

For this report, 26 first patients of the SYNCOPE trial were included to report the feasibility and baseline performance of ctDNA detection. Lymph node invasion was observed in the MRI scans of all patients, except for two. Eleven out of 26 (42.3%) of the patients had T3 tumor and 15/26 (57.7%) T4 tumor. This analysis focused on primary tumor sequencing, baseline plasma ctDNA status, and pre‐treatment variables such as age, sex, and initial clinical TNM staging.

Prospectively performed NGS of the primary tumors allowed us to identify somatic mutations in all 26 cases of the validation cohort with the same panel as used in the development cohort, with the only difference of sequencing DNA from pre‐therapy treatment‐naïve tumors only. A total of 65 mutations were detected (Table S2), with 50 unique variants observed across the 26 primary tumors, affecting 12 genes (Figure S4A). On average, the panel detected 2.5 mutations (range: 1–6) per tumor.

Analyzing prospectively the baseline plasma samples during the trial, detectable ctDNA was found in 80.8% (21 out of 26) of the patients, while the remaining five out of 26 remained ctDNA negative for 1–2 target variants analyzed. All baseline ctDNA negative patients had 2–3 variants detected from the primary tumor, but for three of them, the ctDNA detection relied on a single variant call. In 10 of all 26 patients, ctDNA detection relied on a single target mutation. Of these patients, five had only one mutation found from the primary tumor, whereas the rest five patients had two to six mutations detected from the primary tumor. We observed ctDNA in 70.0% (7/10) of patients with single variant call, compared to 87.5% (14/16) when two variants were targeted. Mutated genes in the baseline ctDNA samples are presented in Figure S4B.

In the validation cohort, baseline ctDNA was positive in 50.0% (1/2) of cII‐stage and 83.3% (20/24) of cIII‐stage rectal cancers (Figure 4B). Positive ctDNA was found in 72.7% (8/11) of T3‐stage and 86.7% (13/15) of T4 rectal cancers (Figure 4C). The combination of baseline ctDNA status and N‐status is depicted in Figure 4D, showing cN2‐tumor with the highest frequency (85.0%, 17/20 for cN2‐, 75.0%, 3/4 for cN1‐ and 50.0%, 1/2 for cN0‐tumors) of baseline plasma ctDNA present.

## 4 Discussion

In this practical implementation study with a clinical trial validation, we aimed to introduce a realistic in‐house ctDNA test to clinical workflow and assess the performance to detect baseline ctDNA with a targeted plasma NGS and/or ddPCR from unselected localized rectal cancers. The tumor‐informed ddPCR test was positive for ctDNA in 58.5% of the development cohort of 41 patients analyzed retrospectively and 80.8% in the validation cohort of 26 patients analyzed prospectively. The validation cohort patients included more locally advanced cancers, which is why it is not surprising that the proportion of ctDNA‐positive patients was higher than in the development cohort. In the development cohort, ddPCR was superior to the NGS panel for detecting ctDNA in baseline plasma samples.

Detectable level of ctDNA was found in baseline plasma more often in clinical stage II‐III cancers than in cI‐cancers, as other studies have reported [26, 37]. Although no difference between cN0‐ and cN1‐2 cancer ctDNA positivity was found in the development cohort when the radiological nodal status was assessed on preoperative MRI, the validation cohort showed that ctDNA was more often positive in cN2 cancers. The lack of difference in the development cohort may be due to the insensitivity of rectal cancer MRI to detect lymph node invasion, and therefore the (y)pTNM‐staging is a more precise measure of true lymph node involvement. The other plausible explanation is that the neoadjuvant therapy‐exposed tumors may have lost tumor cellularity in the development cohort or their mutational profile might have changed as a result of therapy. As a clinical application, a practical baseline ctDNA test could help interpreting the radiological local advancement of rectal cancer, especially considering the known uncertainty of the clinical local lymph node status assessment [38, 39]. ctDNA detection rate in early‐stage cancers presented very high (66.7% for (y)pI‐stage cancers). Problematically, in neoadjuvant treated rectal cancer, the (y)pTNM‐stage is not directly comparable to baseline ctDNA status due to the time gap and likely down‐staging between the baseline sample collection and surgical specimen.

Our baseline ctDNA detection rates in the development cohort indicate slightly inferior results than previously described baseline samples in locally advanced rectal cancers (74%–83%), which might be due to including some early‐stage cancers in this study [29, 30, 40]. Still, in a subgroup analysis, stage II–III cancers at baseline showed similar detection rates close to 83% as previously, although lower than the 90% level that the modern but more expensive commercial multimodal tests have shown [27]. However, most previous studies reporting high ctDNA sensitivities in detecting early‐stage CRC have not reported rectal cancer specifically, but the CRC cohorts have mainly consisted of colon cancer. A distinct feature of rectal cancer is that the decision‐making at baseline is based on therapy‐naïve imaging data points, but the final pathology results are reported 4–6 months later after neoadjuvant therapies have fundamentally altered the local spread of the tumor, making it substantially more difficult to assess the association of clinicopathological characteristics and the presence of ctDNA positivity.

Over one‐third (16/41) of patients were baseline ctDNA negative with ddPCR and/or NGS panel. One possible explanation why 30% (6/20) of (y)pIII‐stage cancers were baseline ctDNA‐negative is the limited release of ctDNA at the time of diagnosis, particularly for the three cases initially diagnosed as stage I cancers and one as stage II. Five of the baseline ctDNA negative patients should have been assumed to be ctDNA positive at baseline as initially clinically interpreted stage III cancers. The rest of the ctDNA‐negative patients (*n* = 5) were early‐stage cancers (3 cI‐ and 2 cII‐stage) cancers, which might be the reason why there was a limited shedding of ctDNA in the baseline. Nevertheless, these results indicate that even seemingly local disease often has adequate levels of ctDNA in serum rendering detection of ctDNA from plasma.

The validation cohort ctDNA detection performance at baseline is similar to previously published results in CRC [21, 37]. Based on ddPCR results alone, more samples in the validation cohort tested positive for ctDNA (81% vs. 58.5%). It is noteworthy that all five of the baseline ctDNA‐negative patients had 2–3 variants identified in their primary tumors, which could have been utilized for ctDNA detection, but for logistical reasons in the clinical laboratory, were not, as it was at the discretion of the laboratory provider to select the ddPCR probes/primers for targeting. This suggests the potential utility of incorporating all available variants from the primary tumor for enhanced ctDNA detection, especially in a narrow‐targeting assay such as ours. Full data of the validation cohort will reach maturity during the ongoing trial, and the effect of comprehensive use of the target variants may contribute to increased sensitivity.

Follow‐up plasma sample with positive ctDNA result predicted recurrence for one patient (#17) in the longitudinal development cohort. Three patients (#19, #25, and #28) should assumingly have had detectable ctDNA in the follow‐up plasma due to metastatic disease and without any ongoing oncological treatment at the time of plasma collection, but the assays showed negative for ctDNA. Notably, two of these patients (#19 and #28) were also baseline ctDNA‐negative with only one variant called from plasma with ddPCR even though 4–5 variants were detected from the primary tumor. On the other hand, neither the NGS panel detected any of the variants present in the primary tumor, indicating true negativity of the plasma in these individuals. For the rest of the follow‐up ctDNA‐negative cases, negative results can be due to ongoing oncological therapies (#7 and #29), a long delay between sample collection and recurrence (#2 and #31), and successful resection of the metastases (#30). The remaining three patients with ctDNA‐positive follow‐up samples remained disease‐free in the routine follow‐up. Due to the short half‐life of ctDNA in plasma [16], we would have expected that all patients with ctDNA in the follow‐up plasma would have had a recurrence at the time of sample collection or shortly after that. However, although the recurrence risk for ctDNA‐positive molecular residual disease is high, it is not 100% [27], and especially local recurrences may be detected with long intervals from primary resection. It must also be noted that there is no certainty that a metastatic rectal cancer should always have detectable amounts of ctDNA in circulation. It is likewise plausible, although unlikely, that a tumor‐informed ctDNA assay using only single markers could yield a false‐positive result, or tumor DNA from a metachronous cancer.

The presence of possible clonal hematopoiesis poses challenges that necessitate careful interpretation, because patients with clonal hematopoiesis often harbor mutations in genes commonly associated with hematopoietic conditions. For example, in patient #14, a primary tumor JAK2 Val617Phe variant was detected both in the baseline and follow‐up plasma sample as the only detected pathogenic variant with high VAF (Table S1). As this particular variant is common for hematological malignancies such as polycythemia vera, it is possible that clonal hematopoiesis may have contributed to the result of this given patient as the patient also had polycythemia vera. However, we did not detect any excessive lymphocytic infiltration in the tumor that would explain the primary tumor sequencing finding.

There are several strengths that highlight the importance of the current report. We report two separate datasets that have been meticulously curated by rectal surgeons and pathologists from the clinical data recorded. The patient material was quite heterogeneous and reflects the real‐life setting, with various cTNM‐stage cancers, mrEMVI‐positive and ‐negative patients, and patients with various neoadjuvant compositions. The study also focused on localized rectal cancers that are less studied for ctDNA status, for which only a few studies exist so far [29, 30, 40]. None of the previous studies have investigated rectal cancer ctDNA detection with several different methods side by side.

There are several limitations to acknowledge. The small sample sizes of both study cohorts led to low statistical power, thereby reducing the likelihood of identifying true associations between ctDNA positivity and oncological outcomes. Also, the retrospective analysis of the samples in the development cohort was a limitation but was addressed in the validation cohort performed prospectively.

Although we tested most patients positive for ctDNA at baseline, some patients remained ctDNA negative. We believe the primary explanation is the small number of targets in the assay based on the validation cohort. One possible explanation for the lack of ctDNA detection in some plasmas is the contamination of genomic DNA from peripheral blood mononuclear cells, as suggested by the relatively high cfDNA concentrations observed in our samples. However, one of the primary purposes of the study was the practical development of a ctDNA application to become adopted into daily practice. With this in mind, we utilized an in‐house singleplex ddPCR test instead of costly commercial options that lack evidence‐based support for adopting in publicly funded healthcare and that were not yet widely available when the study was designed. We also piloted clinically easily available panel sequencing for the detection of the mutations in primary tumors instead of labor‐intensive whole exome sequencing, leading to a limited number of variant targets for tumor‐informed ctDNA detection, for which in five patients none of the 50 hotspot genes were detected. From the financial perspective, a targeted ddPCR test costs only about 1/10 of the most advanced multimodal cfDNA assays, whereas the sensitivity for detection is not much inferior.

Multiple serial plasma samples would have elaborated knowledge about ctDNA trajectories after different treatments than our two‐timepoint serial analysis. Since the timing of the plasma collection with respect to chemoradiotherapy is of utmost importance, and it is not fully understood how neoadjuvant treatment may affect ctDNA status, we included a post‐neoadjuvant therapy plasma sample collection in the clinical trial NCT04842006 currently enrolling. Once the clinical data matures, we will be able to address the question on the most informative prognostic time point for plasma collection, and whether the mutational profile may change during the neoadjuvant therapy.

## 5 Conclusions

In conclusion, our study and the ongoing trial indicate that ctDNA detection with tumor‐informed oligomarker ddPCR is superior to NGS panel sequencing in patients with non‐metastatic rectal cancer. Additionally, ctDNA was frequently detected in baseline plasma samples of patients with advanced cancers, but not before most recurrences' appearance. Still, large clinical trials should evaluate the clinical usefulness of ctDNA in multidisciplinary rectal cancer team decision‐making, as well as a biomarker of the need for combination chemotherapy such as total neoadjuvant therapy.

## Author Contributions

**Säde Szeto:** data curation (equal), formal analysis (equal), investigation (equal), visualization (equal), writing – original draft (equal), writing – review and editing (equal). **Soili Kytölä:** data curation (equal), investigation (equal), methodology (equal), writing – review and editing (equal). **Erdogan Pekcan Erkan:** software (equal), supervision (equal), writing – review and editing (equal). **Maarit Ahtiainen:** data curation (equal), investigation (equal), methodology (equal), writing – review and editing (equal). **Jukka‐Pekka Mecklin:** resources (equal), writing – review and editing (equal). **Teijo Kuopio:** resources (equal), writing – review and editing (equal). **Ville Sallinen:** resources (equal), writing – review and editing (equal). **Anna Lepistö:** resources (equal), writing – review and editing (equal). **Laura Koskenvuo:** data curation (equal), resources (equal), writing – review and editing (equal). **Laura Renkonen‐Sinisalo:** resources (equal), writing – review and editing (equal). **Anu Anttonen:** resources (equal), writing – review and editing (equal). **Kukka Heiskala:** resources (equal), writing – review and editing (equal). **Sanni Tulokas:** resources (equal), writing – review and editing (equal). **Siru Mäkelä:** resources (equal), writing – review and editing (equal). **Erkki‐Ville Wirta:** resources (equal), writing – review and editing (equal). **Tuija Tuunanen:** resources (equal), writing – review and editing (equal). **Tapio Salminen:** resources (equal), writing – review and editing (equal). **Ari Ristimäki:** investigation (equal), resources (equal), supervision (equal), writing – review and editing (equal). **Toni T. Seppälä:** conceptualization (equal), funding acquisition (equal), project administration (equal), resources (equal), supervision (equal), writing – original draft (equal), writing – review and editing (equal).

## Ethics Statement

The study was approved by the ethical committee of the Central Finland Hospital district and institutional review board (1U/2018 at 1/2019). SYNCOPE trial was approved by the Helsinki and Uusimaa ethical committee study site‐specific institutional review boards and national Finnish Medicines Agency FIMEA (HUS/2427/2020 and HUS approval HUS/155/2021, TAYS approval §131 R22043M, trial registration NCT04842006, EudraCT 2020‐003697‐52).

## Consent

All patients in this study have given their written consent to use their samples and clinical data as a part of this study.

## Conflicts of Interest

T.T. Seppälä reports consultation fees from Tillots Pharma, Nouscom, and Mehiläinen, being a co‐owner and CEO of Healthfund Finland Ltd., and a position in the Clinical Advisory Board and a minor shareholder of LS Cancer Diag Ltd. The other authors declare no conflicts of interest.

## Supporting information


Appendix S1.

## Data Availability

The data supporting the results of this article are available within the article or its Appendix S1. Otherwise, anonymized data and analysis codes are available by contacting the corresponding author with a reasonable request.
