# Supplementary material for: Performance Comparison of Droplet Digital PCR and Next‐Generation Sequencing for Circulating Tumor DNA Detection in Non‐Metastatic Rectal Cancer
Source: Cancer Med. 2025 May 9;14(9):e70943. doi: 10.1002/cam4.70943 (PMC12062871; doi:10.1002/cam4.70943)
Supplement: Supplementary file 1 — Appendix S1. [file CAM4-14-e70943-s001.docx]

**Performance comparison of droplet digital PCR and next generation sequencing for circulating tumor DNA detection in non-metastatic rectal cancer**

Säde Szeto^1^, Soili Kytölä^2, 3^, Erdogan Pekcan Erkan^1, 4^, Maarit Ahtiainen^5^, Jukka-Pekka Mecklin^6, 7^, Teijo Kuopio^5, 8^, Ville Sallinen^9, 10^, Anna Lepistö^9^, Laura Koskenvuo^9^, Laura Renkonen-Sinisalo^9^, Anu Anttonen^11^, Kukka Heiskala^11^, Sanni Tulokas^11^, Siru Mäkelä^11^, Erkki-Ville Wirta^4, 12^, Tuija Tuunanen^13^, Tapio Salminen^13^, Ari Ristimäki^1, 14^, and Toni T. Seppälä ^1, 4, 12^

^1^ Applied Tumor Genomics Research Program, Research Program Unit, Faculty of Medicine, University of Helsinki, Helsinki, Finland

^2^ Department of Genetics, HUS Diagnostic Center, Helsinki University Hospital, Helsinki, Finland

^3^ Department of Genetics, University of Helsinki, Helsinki, Finland

^4^ Faculty of Medicine and Health Technology, Tampere University and Tays Cancer Centre, Tampere University Hospital, Tampere, Finland.

^5^ Department of Molecular Pathology, Central Finland Hospital Nova, Wellbeing Services County of Central Finland, Jyväskylä, Finland

^6^ Department of Education and Science, Central Finland Hospital Nova, Wellbeing Services County of Central Finland, Jyväskylä, Finland

^7^ Faculty of Sports and Health Sciences, University of Jyväskylä, Jyväskylä, Finland

^8^ Department of Biological and Environmental Science, University of Jyväskylä, Jyväskylä, Finland.

^9^ Department of Gastroenterological Surgery, Helsinki University Hospital and University of Helsinki, Helsinki, Finland

^10^ Transplantation and Liver Surgery, Helsinki University Hospital and University of Helsinki, Helsinki, Finland

^11^ Department of Oncology, HUS Comprehensive Cancer Centre and University of Helsinki, Helsinki, Finland

^12^ Department of Gastroenterology and Alimentary Tract Surgery, Tampere University Hospital, Elämänaukio, Tampere, Finland.

^13^ Department of Oncology, Tampere University Hospital, Tampere, Finland

^14^ Department of Pathology, HUS Diagnostic Center, Helsinki University Hospital, Helsinki, Finland

Correspondence

Säde Szeto, Applied Tumor Genomics Research Program, Research Program Unit, Faculty of Medicine, Biomedicum 1, Haartmaninkatu 8, 00014 University of Helsinki, Finland. E-mail: [sade.szeto@helsinki.fi](mailto:sade.szeto@helsinki.fi). ORCID ID: 0009-0006-5603-1860.

Toni T. Seppälä Faculty of Medicine and Health Technology, Tampere University, Kauppi Campus, Arvo Building, B212/D243-244, 33520 Tampere, Finland. E-mail: [toni.seppala@tuni.fi](mailto:toni.seppala@tuni.fi). ORCID ID: <https://orcid.org/0000-0002-4940-3498>.

**Supplementary Materials – Index**

Study design *pag. 4*

| **Supplementary Methods** |  | |  |
| --- | --- | --- | --- |
| DNA isolation from primary tumors and panel sequencing | *pag. 5* |  |  |
| Preanalytical preparation of plasma DNA | *pag. 5* |  |  |
| ctDNA analysis | *pag. 6* |  | |
| **Supplementary Results** |  | |  |
| Primary tumor sequencing results | *pag. 7* | |  |
| Mutational landscape of ctDNA  Baseline cfDNA concentration  Baseline ctDNA status and clinical characteristics | *pag. 8*  *pag. 9*  *pag. 9* | |  |
| Baseline ctDNA analysis with ddPCR  Discrepancy in clinical and pathological TNM-staging  Recurrence-free patients with follow-up samples  Recurrence and survival | *pag. 10*  *pag. 10*  *pag. 12*  *pag. 12* | |  |
| **Supplementary Appendixes** |  | |  |
| Detailed description of recurring patients | *pag. 13* | |  |
|  |  | |  |
| **Supplementary Figures and Tables** |  | |  |
| Supplementary Figure 1 | *pag. 17* | |  |
| Supplementary Figure 2  Supplementary Figure 3  Supplementary Figure 4  Supplementary Table 1  Supplementary Table 2 | *pag. 18*  *pag. 20*  *pag. 21*  *pag. 22*  *pag. 25* | |  |
|  |  | |  |
|  |  | |  |

**1.**  **Supplementary Materials**

Study design

All 46 patients selected for the development cohort had clinical TNM-stage I-III (cTNM) cancer in pre-therapy pelvic magnetic resonance imaging (MRI) at baseline. Metastatic diseases were excluded. Most of the early-stage cancer (cI-II-stage) patients had at least one high-risk feature, e.g., T4-growth in MRI or extramural vascular invasion in MRI (mrEMVI+). The decision of neoadjuvant treatment was made by a multi-professional group based on tumors clinicopathological features. The patients guided to neoadjuvant therapies, underwent eighter a short radiotherapy (5x5 Gy) or long-course 50.4/1.8 Gy radiotherapy with capecitabine radiosensitizer for neoadjuvant treatment.

The validation cohort included pre-therapy tumor tissue specimens and baseline plasma samples from first 26 patients with local rectal cancer randomized to the SYNCOPE trial. The intervention arm patients underwent total neoadjuvant therapy with 5x5 Gy radiotherapy and four cycles of capecitabine-oxaliplatin (CAPOX) combination chemotherapy. Patients randomized to conventional group received 28x1.8 Gy radiotherapy with capecitabine radiosensitizer. All patients were mrEMVI+ at baseline, as it was one of the inclusion criteria for the SYNCOPE trial.

All patients recruited for this study were planned curative-indented surgery. The decision on adjuvant therapy was made by a multi-professional team based on their evaluation of patients` recurrence risk in the development cohort without additional information from the ctDNA. For the validation cohort, the decision strategy of adjuvant treatments will depend on the randomization group (not assessed in this study).

Baseline carcinoembryonic antigen (CEA) concentrations were defined from the blood samples drawn at the same time point as baseline ctDNA. Computer tomography (CT) scans for follow-up were screened by surveillance protocol, roughly one and two years after the operation.

**2.**  **Supplementary Methods**

2.1 DNA isolation from primary tumors and panel sequencing

Primary tumor tissue specimens were collected and prepared as a formalin-fixed paraffin-embedded tissue (FFPE) block according to standard protocol. The pathologist defined a representative area of the sample with a substantial number of neoplastic cells. Samples were sent to an accredited laboratory in HUSLAB (Helsinki, Finland) for purchased panel sequencing, by the next generation sequencing (NGS) -technique. Before sequencing, DNA was isolated from the FFPE samples by Qiagen FFPE Kit (Qiagen cat #56494). At a minimum, 10ng of DNA was used in mutation identification. Torrent Suite Software and Ion Reporter Software were used to analyze NGS-panel-sequencing data for variant calls.

## 2.2 Preanalytical preparation of plasma DNA

The blood samples were processed according to the approved standard operating procedure (SOP) for blood collection and handling (<https://www.streck.com/wp-content/uploads/sync/Stabilization/Cell-Free_DNA_BCT_RUO_CE/02_Product_Information/06_Isolation_of_WBCs_collected_from_Cell-Free_DNA_BCT_and_Nucleic_Acid_BCT.pdf>).

Briefly, plasma was isolated from whole blood by double centrifugation (two rounds of 1600 g for 10 minutes). Aliquots of plasma were stored at -80˚C until shipment or extraction of cfDNA. cfDNA was isolated from 3-4 ml of plasma using the Qiasymphony SP with Qiagen QIAamp Circulating Nucleic Acids Kit (Qiagen GmbH 937236, Qiagen Strasse 1, 40724 Hilden, Germany) and eluted in LoBind tubes (Eppendorf. AG). The concentration of cfDNA was assessed using the Qubit Fluorometer (Thermo Fisher Scientific Inc.)

2.3 ctDNA analysis

Droplet Digital PCR (ddPCR) by BioRad (www.biorad.com) was used for ctDNA detection in extremely low variant allele frequencies (VAFs). BioRad designed the one to two probes for ddPCR primarily based on the primary tumor mutations with the highest allele frequencies. Whenever feasible, two probes were utilized in the ddPCR analysis. However, for some only one probe was utilized due to the probe availability. Custom probe ordering was avoided to minimize the expenses and significant delays in our analysis. We used 2-9µl extracted DNA for each triplicate reaction, and the QX200 Droplet Generator separated samples into 20 000 droplets for PCR amplification. 2µl extracted DNA for the development group and 9 µl for the validation group was assessed. After the amplification, the QX200 Droplet reader analyzed the droplets individually in a thermocycler. The absolute quantity of targeted DNA was calculated based on PCR-positive and PCR-negative droplets and converted into digital form. At least two positive droplets were required to classify a sample as positive.

To establish the VAF threshold for ctDNA positivity with the NGS panel, we manually reviewed baseline variants with VAF <1% from raw data for the first 20 patients (1–20 in Supplementary Table 1) in the development group. These mutations were not detected in variant calls as the threshold with NGS panel is VAF ≥1% for a variant call. For these patients, variants detected in the primary tumor and baseline plasma with VAFs > 0.01% but <1% were also interpreted as positive results by NGS panel. For the remaining 21 patients (21–41 in Supplementary Table 1) in the development group, the results were not manually viewed and only variants detected in the variant calls were interpret as positive (VAF threshold ≥ 1%). Consequently, we defined a threshold of 0.01% for the NGS panel, as approximately half of the patients also baseline variants with VAFs > 0.01% but <1% were interpreted as positive results.

NGS-panel sequencing and ddPCR services were purchased from an accredited laboratory (T055; qualifications SFS-EN ISO/IEC 17025:2005 and SFS-EN ISO 15189:2013) in HUSLAB and performed by a clinical geneticist.

**3.**  **Supplementary Results**

3.1 Primary tumor sequencing results

The primary tumor sequencing results for the five patients without detectable mutations are unlikely to be attributed by neoadjuvant therapies because only one patient out of five received neoadjuvant treatments. All mutated genes in the primary tumors of the development cohort are presented in **Supplementary Figure 2C**. VAF of mutations identified, ranged from 3.0% to 76%, with a median VAF of 27%.

In the validation group, *TP53* (30.8%, 20/65), *APC* (20.0%, 13/65), and *KRAS* (13.8%, 9/65) genes were most frequently mutated in the primary tumor sequencing (**Supplementary Figure 4A)**. The median VAF was 18%, ranging from 4% to 68% in the validation cohort.

In the development cohort, the primary tumor mutations VAF correlated with tumor cellularity reported in the final pathological examination (*p*=0.00015, **Supplementary Figure 2A**). High VAF in the tumors` mutation was associated with patients without recurrence (*p*=0.011, **Supplementary Figure 2B**), but not with baseline ctDNA positivity (*p*=0.69, **Supplementary Figure 2E)**.

3.2 Mutational landscape of ctDNA

Genes that were affected by mutations observed in the development group are presented in **Supplementary Figure 2D**. The mean VAF for baseline ctDNA mutations was 1.0% (range 0.010 to 27%) with NGS-panel and 0.80% (range 0.12 to 33%) for ddPCR.

Based on our analysis, only five different genes had mutations in the follow-up plasmas of development group patients: *APC* (28.6%, 2/7), *TP53* (28.6%, 2/7), *JAK2* (14.3%, 1/7), *NRAS* (14.3%, 1/7), and *SMAD4* (14.3%, 1/7). All of these were identified from four patients’ samples and one of them had four different genes mutated in the follow-up plasma. The median VAF was 0.56% (range 0.28 to 70%) with NGS and 1.0% (range 0.50 to 72%) with ddPCR.

In the validation cohort, the most frequently queried and detected genes in baseline plasma were *TP53* (37.5%, 12/32), *APC* (21.9%, 7/32), and *KRAS* (12.5%, 4/32) (**Supplementary Figure 4B).** In terms of VAF of the identified mutations, the median VAF was 1.6%, ranging from 0.24% to 7.3%.

All variants detected in the primary tumor- and plasma samples and their precise allele frequencies are listed in **Supplementary Table 1** for the development group and in **Supplementary Table 2** for the validation group.

3.3 Baseline cfDNA concentration

cfDNA concentration was not normally distributed (*p*= 0.00087, Shapiro-Wilk test). Higher cfDNA concentrations were not associated with either sex (*p*=0.85), cTNM-stages (*p*=0.22), cN-status (*p*=0.46), (y)pTNM-stages (*p*=0.34), mrEMVI-status (*p*=0.42), (y)pEMVI-status (*p*=0.60), baseline ctDNA-status (*p*=0.45) and recurrences (*p*=0.42). Notably, when cTNM-stage was observed as groups, stage II and III cancers had higher cfDNA concentrations than stage I cancers (*p*=0.046, two-tailed Fishers exact test). Still, differences in cfDNA concentration based on grouped (y)pTNM-stage (*p*=0.31) and grouped cN-status (lymph node invasion in initial MRI) (*p*=0.34) remained statistically non-significant. The average cfDNA concentrations in most relevant clinicopathological variables are presented in **Supplementary Figure 1.**

3.4 Baseline ctDNA status and clinical characteristics

Nearly two-thirds (64.3%; 9/14) of (y)pN1-2 and baseline ctDNA positive patients were cN1-2 initially, of which eight received neoadjuvant therapies. Only one-third (36.4%; 4/11) of (y)pN0- and baseline ctDNA positive cancers were cN1-2 originally, of which three patients received chemoradiotherapy before surgery. Four out of six baseline ctDNA negative (y)pN1-2 cancers were originally cI-II stage cancers, that did not undergo neoadjuvant therapy. Five out of ten (y)pN0 and baseline ctDNA negative patients had cI-II stage cancer and none underwent neoadjuvant therapy. Moreover, 57.9% (11/19) of cN0 and mrEMVI- patients were ctDNA positive at baseline.

No difference with age (*p* =0.34), sex (*p* =1.0), cfDNA concentration (*p*=0.90), cN-status (*p* =0.75), (y)pT-status (*p*=0.72), (y)pN-status (*p*=0.34), (y)pEMVI (*p*=1.00), ypTRG (*p*=1.00) or lymph node yield (*p*=0.22) between baseline ctDNA positive and negative patients was detected.

3.5 Baseline ctDNA analysis with ddPCR

The detection rates for ctDNA combined with ddPCR and NGS-panel is presented in the main text. In the development group, ddPCR detected ctDNA from 24 out of 41 baseline plasma samples. Baseline ctDNA detection rates were 14.3% (1/7), 71.4% (10/14), and 65.0% (13/20) for cI, cII, and cIII stage cancers by ddPCR, respectively. When ddPCR -analysis was used unattended, ctDNA was detected from the baseline plasma from 14.3% of cI-stage cancers compared to 67.6% plasma from cII-III-stage cancers (*p*=0.014). ddPCR performance for baseline ctDNA detection in T1-2 cancer was 22.2% (2/9) and in more advanced cT3-4 cancers 68.8% (22/32) (*p*=0.021).

Based on the type of mutation used as probes, the detection rates for ctDNA were 52.0% (13/25) for *TP53*, 30.8% (4/18) for *KRAS*, 36.4% (4/11) for *APC*, 66.7% (2/3) for *NRAS*, 66.7 % (2/3) for *BRAF*, 50.0% (1/2) *GNAS*, 100.0% (1/1) for *JAK2* and 0.0% (0/1) for *PIK3CA* in the development group*.* For the validation cohort, the detection rates were 80% (12/15) for *TP53*, 50% (4/8) for *KRAS*, 100% (7/7) for *APC*, 75% (3/4) for *NRAS*, 100% (3/3) for *FBXW7*, 100% (2/2) for *PIK3CA*, 100% (1/1) for *BRAF* and 0% (0/1) for *MET*.

3.6 Discrepancy in clinical and pathological TNM-staging

Among the patients included in the study, 13 exhibited an upstaging from cTNM to (y)pTNM staging. Notably, nine of these patients did not receive neoadjuvant therapy, comprising five patients with cI-stage cancer and 4 with cII-stage cancer, which progressed to two (y)pII-stage and seven (y)pIII-stage cancers, respectively. Four patients experienced tumor upstaging despite receiving neoadjuvant therapy, of which two were treated with short-course radiotherapy (from cII- to (y)pIII-stage) and two with chemoradiotherapy (from cIII- to (y)pIV-stage). Among the subgroup of patients who experienced tumor upstaging (n=13), seven out of eleven (63.6 %) (y)pIII-IV cancers were positive for baseline ctDNA.

Sixteen patients exhibited consistent clinical staging between the initial assessment and the pathological specimen, with two cases classified as stage I, five as stage II, and nine as stage III. Seven of the patients received neoadjuvant therapies, of which all except one was stage III cancer. Among the stage III cancers, seven out of nine (77.8 %) cases were positive for baseline ctDNA, while three out of five (60.0 %) stage II cancers exhibited ctDNA presence in baseline plasma.

Twelve patients demonstrated downstaging as the tumor was considered higher cTNM-stage than the TNM-staging in the final pathological examination. 41.7 % (5/12) of these patients did not receive neoadjuvant therapy, indicating unsuccessful staging through radiological assessment. Among these patients, four out of five had ctDNA in the baseline sample, with only one (y)pII-stage cancer non-detectable ctDNA in the baseline plasma. Interestingly, the remaining three patients had (y)pI-stage cancer and exhibited ctDNA presence in the baseline plasma. In contrast, among the seven cIII-stage cancers that received neoadjuvant therapy, pathological evaluation downstaged these tumors to stage I-II, indicating successful neoadjuvant therapy. Only three of these seven patients were ctDNA positive at baseline.

Among the cohort of 41 patients, 34.1% (14/41) of the patients were identified to have MRI-confirmed extramural vascular invasion (mrEMVI+). Among these patients, 50.0% (7/14) also exhibited pathologically confirmed vascular invasion ((y)pEMVI+). All except one mrEMVI+ patients received neoadjuvant therapy, which might explain why half of the patients lacked vascular invasion in the final pathological assessment. Interestingly, only 38.9 % (7/18) of the patients with pathologically confirmed vascular invasion were also mrEMVI+ and all these patients underwent neoadjuvant therapy. Furthermore, four out of the eleven mrEMVI- and (y)pEMVI+ patients underwent neoadjuvant therapy. The discrepancy of TNM-staging in clinical and pathological assessment combined with ctDNA-status and treatment interventions is illustrated in **Figure 1B**.

3.7 Recurrence-free patients with follow-up samples

Twenty-five follow-up plasma samples were analyzed, of which only four were ctDNA positive, when NGS and ddPCR were both utilized. Of these patients, only one had a recurrence while three patients remained recurrence-free. Two of these recurrence-free patients had cII- and one cIII-stage cancer and in the final pathological analysis both cII cancers were considered as (y)pIII tumors and the cIII was considered as pII tumor. Only one cII cancer patient received neoadjuvant and adjuvant treatment other two patients did not receive (neo)adjuvant therapies. The follow-up plasma samples of these three patients were collected on average 13 months after operation. **Supplementary Figure 3** represents recurrence-free patients with follow-up samples patients in detail. For all recurrence-free patients with follow-up samples, the average time from the latest CT scan or colonoscopy was 31 months and 47 months after the operation.

3.8 Recurrence and survival

The follow-up times from the operation to the latest colonoscopy or CT scan are skewed shorter due to loss of follow-up for patients who live outside the catchment area (n=13). Therefore, the end of clinical follow-up was defined as the last follow-up at the Meilahti hospital. Information on survival and recurrence were complete for all patients.

Only four out of ten patients with recurrence received adjuvant therapy to reduce the risk of recurrence. One of these patients relapsed during the adjuvant therapy (#7). Two of the patients did not receive the total adjuvant therapy: one patient received only half of the adjuvant therapy (#17) and the other only one cycle (#25) leading to insufficient risk reduction.

**4.**  **Supplementary Appendixes**

Detailed description of recurring patients

Patient #2 had stage II cancer and neoadjuvant therapy was not given. Both baseline and follow-up plasma were ctDNA negative. The follow-up sample was taken roughly 16 months after the operation and the patient was diagnosed with lung metastasis in the surveillance CT scan roughly 49 months after the operation. The 33-month interval between the collection of follow-up sample and recurrence explains why ctDNA was not detected in the sample. Currently, chemotherapy is considered for the treatment of metastatic disease.

Patient #3 had cIII-stage cancer and ctDNA was detected from baseline plasma. The patient underwent chemoradiotherapy before surgery. Malignant-suspect masses in the liver were already suspected during the operation and later confirmed as metastases in the CT scan and biopsies. (y)pIV-stage tumor was resected. The patient received oncological therapies for metastatic disease and follow-up plasma was not collected. Due to the patient living outside the catchment area, the stability or progression of the metastatic disease is unclear, but the patient remains alive.

Patient #7 had cIII-stage cancer but ctDNA was not detected from baseline plasma. The patient received long chemoradiotherapy and (y)pII-stage cancer was resected. Only one month after the operation, liver metastases were detected during adjuvant therapy in the response CT. Liver metastases were operated four months after recurrence. The follow-up ctDNA was measured during adjuvant chemotherapy and roughly two months after liver metastasectomy, which might explain ctDNA negativity in the follow-up plasma. Oncological therapy continued after the operation, but a new inoperable metastasis was detected in the lungs 16 months after the primary tumor resection, and the patient was referred to palliative care.

Patient #11 had cIII-stage cancer and baseline ctDNA was positive. The patient was treated with chemoradiotherapy before the operation and (y)pII-stage rectal cancer was resected. The patient did not receive any adjuvant therapy, and 14.5 months after the operation patient was diagnosed with a tumor mass in the iliac lymph nodes at surveillance CT, which was considered a residual tumor. Oncological therapy was recommended by the multidisciplinary team for treatment. Follow-up plasma was not collected. Due to the patient living outside the catchment area, the stability or progression of the metastatic disease is unclear, but the patient remains alive.

Patient #17 had cIII-stage cancer and ctDNA was positive at baseline. The patient underwent chemoradiotherapy and (y)pIII-stage cancer was resected. Only half of the adjuvant therapy was accomplished due to side effects. Only 4 months after adjuvant therapy was discontinued, liver metastasis was discovered and treated with chemotherapy and metastasectomy. The follow-up plasma which contained ctDNA, was taken one month before the diagnosis of liver metastasis, without any ongoing adjuvant treatments. The patient died roughly 2.5 years after the operation due to widespread cancer.

Patient #19 had cIII-stage cancer with negative baseline ctDNA. The patient underwent chemoradiotherapy before surgery, and (y)pII-stage rectal cancer was resected. Roughly one year after the operation, lung metastasis was detected despite complete adjuvant chemotherapy being given. Lung metastases were operated with a 14-month delay without any oncological therapy. Still, the patient has remained disease-free without further oncological therapy after the operation. The follow-up plasma was collected at the time of lung metastasis and found surprisingly ctDNA negative despite the metastatic disease.

Patient #25 had cII-stage cancer, and the baseline plasma contained ctDNA. (y)pIII-stage cancer was resected directly after the diagnosis. The patient received only one cycle of capecitabine due to severe side effects of the cytostatic agent. At 17 months after the operation, a lung metastasis was detected in the surveillance CT. The follow-up plasma was taken only one month after recurrence, and it was surprisingly negative, despite the lack of oncological therapies by the time of sample collection. The lung metastasis was treated with radiotherapy, but five months later, liver metastases were also detected. Other oncological therapies were also continued, but eventually, the patient was assigned to palliative care.

Patient #28 was diagnosed with cI-stage cancer, which was operated directly. Baseline ctDNA was negative despite, being classified as an (y)pIII-stage tumor in the pathological examination. Adjuvant chemotherapy was not considered. Six months after the surgery, a CT scan revealed a nodule in the lungs and later confirmed as a lung metastasis. Oncological therapies were abstained due to the fragility of the patient. The follow-up plasma was taken six months after the recurrence and found negative. The metastatic cancer progressed to the brain and the patient died to cancer two years after diagnosis.

Patient #29 was diagnosed with III-stage cancer in the clinical and pathological examination. The baseline ctDNA was positive. Despite stage III cancer, preoperative treatments and adjuvant chemotherapy were refrained. Roughly one month after the surgery a CT scan exposed a nodule in the lungs, which was considered as a lung metastasis. The metastasis was operated seven months after being discovered and continued with chemotherapy for three months. The follow-up sample was negative and taken 8.5 months after recurrence; with ongoing chemotherapy. Nineteen months after the primary operation, local cancer progression was observed and treated with further chemotherapy with beneficial response. During the surveillance, the cancer has not indicated any signs of activation.

Patient #30 had cIII-stage cancer and ctDNA was positive at baseline. The patient underwent chemoradiotherapy, which was discontinued due to a coronary spasm. In the preoperative response imaging only a few days before the primary tumor resection, multiple liver metastases were noticed. (y)pIV-stage cancer was resected 4.5 months after the diagnosis. The liver metastases were treated with chemotherapy and liver metastasectomy was performed nine months after diagnosis. Despite not receiving adjuvant therapy, the patient remained disease-free, which may explain the negative follow-up ctDNA 10 months after the operation.

Patient #31 had cII-stage cancer with ctDNA detected at baseline plasma. The patient received long chemoradiotherapy and (y)pII-stage tumor was resected. To prevent recurrence, the patient was treated with adjuvant chemotherapy. Eleven months after adjuvant therapy, follow-up plasma was negative for ctDNA. Still, roughly one year after the follow-up sample collection, the patient was revealed to have multiple lung- and lymph node metastases. Despite multiple regimens of oncological therapy, the cancer progressed, and the patient died to widespread cancer eight months after the recurrence. Neither cTNM-staging nor (y)pTNM-staging or follow-up ctDNA-status were predictive for this disease-related death. Certainly, all these estimates were evaluated with extensive periods with the recurrence.

Patient #41 had cIII-stage cancer and ctDNA was detected in the baseline plasma. The patient received long chemoradiotherapy and the tumor shrunk down to an (y)pII-stage tumor. Adjuvant therapy was omitted and 11 months after the operation, liver metastases were detected in a CT scan. The patient received chemotherapy for liver metastases, but four months after recurrence, also leptomeningeal metastases were observed. None of the metastases were resectable and the patient died three years after the operation to a widespread cancer. Follow-up plasma was not collected.

**4.**  **Supplementary Figures and Tables**

**Supplementary Figure 1** Comparative analysis of baseline cfDNA concentration across clinical parameters.

**A)** A difference in cfDNA concentration between males and females was not distinguished (*p*=0.85, Mann-Whitney rank sum test).

**B)** Differences in cfDNA concentrations across clinical TNM-stages were not detected (*p*=0.22, Kruskal-Wallis test).

**C)** Clinical TNM-stage II-III cancers had statistically higher cfDNA concentrations at baseline than cI stage cancers (*p*=0.046, Mann-Whitney rank sum test).

**D**) Greater quantities of cfDNA were not released in patients with mrEMVI-positive patients compared to mrEMVI-negative patients (*p*=0.42, Mann-Whitney rank sum test).

**E)** Baseline ctDNA positive patients did not have higher cfDNA concentrations compared to baseline ctDNA negative patients (*p*=0.45, Mann-Whitney rank sum test).

**F)** Patients with recurrence had no remarkably higher baseline cfDNA concentrations in comparison to disease-free patients (*p*=0.42, Mann-Whitney rank sum test).

cfDNA, cell-free DNA; cTNM, initial clinical TNM staging; mrEMVI, extramural vascular invasion; ctDNA, circulating tumor DNA.

**Supplementary Figure 2** Mutational landscape in tumor and plasmas.

1. In the development group, a statistically significant correlation between the patient’s tumor cellularity and variant allele frequency in the tumor tissue specimen was observed (*p*=0.00015, Pearson correlation coefficient).
2. Disease-free patients had higher VAF in the mutations found from the primary tumor than patients with recurrence (*p*=0.011, Mann-Whitney test).
3. From 101 identified variants, the mutations located in *TP53* (33.7%), *APC (30.7*%), *KRAS* (14.9%), *FBXW7* (5.9%), *BRAF* (4.0%), *GNAS* (3.0%), *NRAS* (3.0%), *PIK3CA* (2.0%), *SMAD4* (2.0%), and *JAK2* (0.99%) primary tumors genes in the development cohort.
4. Forty mutations were detected in *TP53* (42.5%), *APC* (20.0%), *KRAS* (17.5%), *NRAS* (7.5%), *BRAF* (5.0%,), *GNAS* (2.5%), *JAK2* (2.5%) and *SMAD4* (2.5%) genes in baseline ctDNA of the development cohort.
5. A statistically significant difference between tumors` VAFs between baseline ctDNA positive- and negative patients was not discovered in the development cohort.

ctDNA, circulating tumor DNA; VAF, variant allele frequency.

**Supplementary Figure 3** A swim track plot of ctDNA-status, TNM-stages and treatment interventions of the disease-free patients with follow-up plasma samples. One patient (#14) had inconsistent nodules in the lungs already at the time of diagnosis, but the nodules remained stable in the surveillance CT scans. The follow-up by protocol has already ended signified as the latest CT scan was taken two years after the surgical prevention. These findings were considered as normal findings despite biopsy was never collected. One patient (#22) had a mutation in the baseline and follow-up plasma that was not present in the primary tumor. This *TP53 Arg273*-variant was detected with that same HS1 panel that was used for primary tumor sequencing. One patient (#33) died to non-cancer-related reasons.

**Supplementary Figure 4** Mutational landscape in tumor and plasmas of validation cohort.

1. In the validation cohort, 65 mutations were identified, of which the mutations located in *TP53* (30.8%), *APC* (20.0%), *KRAS* (13.9%), *FBXW7* (9.2%), *NRAS* (9.2%), *PIK3CA* (4.6%), *PTEN* (4.6%), *BRAF* (1.5%), *MET* (1.5%), *PTPN11* (1.5%), *SMAD4* (1.5%), and *SMARCB1* (1.5%) genes.
2. Thirty-two mutations were detected in baseline ctDNA, of which they located in *TP53* (37.5%), *APC* (21.9%), *KRAS* (12.5%), FBXW7 (9.4%), *NRAS* (9.4%), *PIK3CA* (6.3%) and *BRAF* (3.1%) genes in the validation group.

**Supplementary Table 1** All variants detected in tumor tissue of the development cohort group, baseline- and follow-up plasmas, and their precise variant allele frequencies.

|  |  |  |  |  |  |  |  |  |  |  | |  |  |  |  | | | | |
| --- | --- | --- | --- | --- | --- | --- | --- | --- | --- | --- | --- | --- | --- | --- | --- | --- | --- | --- | --- |
|  | **Patient** | **Primary tumor** |  | **NT** |  | **Baseline ctDNA** |  |  | **Other treatments** |  | **Follow-up ctDNA** | | | | |  |  |  |  |
|  |  | **Variant** | **VAF** |  | **ddPCR** | **VAF** | **NGS** | **VAF** |  | **ddPCR** | | **VAF** | **NGS** | **VAF** |  | | | | |
|  |  | **GNAS Arg201His** | 63 % |  | Neg | 0 % | Neg | 0 % |  |  | |  |  |  |  | | | | |
|  | 1 | **BRAF Val600Glu** | 37 % | CRT | Pos | 0.7 % | Neg | 0 % | Surgery |  | |  |  |  |  | | | | |
|  |  | APC Arg1114Ter | 37 % |  |  |  | Neg |  | Adjuvant therapy |  | |  |  |  |  | | | | |
|  |  | APC Glu1554Ter | 28 % |  |  |  | Neg |  |  |  | |  |  |  |  | | | | |
|  | 2 | **TP53 Arg248Gln** | 29 % |  | Neg | 0 % | Neg | 0 % | Surgery | Neg | | 0 % | Neg | 0 % |  | | | | |
|  | 3 | **TP53 Arg342Ter** | 14 % | CRT | Pos | - | Pos | 0.39 % | Surgery |  | |  |  |  |  | | | | |
|  |  | APC Thr1493Argfs*14 | 12 % |  |  |  | Neg | 0 % | Adjuvant therapy |  | |  |  |  |  | | | | |
|  |  | **TP53 Arg175His** | 19 % |  | Neg | 0 % | Neg | 0 % | Surgery | Neg | | 0 % | Neg | 0 % |  | | | | |
|  | 4 | **KRAS Gly12Val** | 15 % | RT | Neg | 0 % | Neg | 0 % | Adjuvant therapy | Neg | | 0 % | Neg | 0 % |  | | | | |
|  |  | SMAD4 Gly508Asp | 12 % |  |  |  | Neg | 0 % |  |  | |  | Neg | 0 % |  | | | | |
|  | 5 | **APC Glu1309Aspfs*4** | 40 % | RT | Pos | 1.0 % | Pos | 0.01 % | Surgery | Pos | | 0.5 % | Neg | 0 % |  | | | | |
|  |  | **TP53 Tyr163Asn** | 27 % |  | Pos | 0.4 % | Pos | 0.39 % | Adjuvant therapy | Neg | | 0 % | Neg | 0 % |  | | | | |
|  | 6 | **KRAS Gly12Ala** | 13 % | CRT | Pos | 0.7 % | Pos | 1.0 % | Surgery |  | |  |  |  |  | | | | |
|  |  | **APC Glu1309Aspfs*4** | 13 % |  | Neg | 0 % | Neg | 0 % |  |  | |  |  |  |  | | | | |
|  |  | **KRAS Gly13Asp** | 10 % |  | Neg | 0 % | Neg | 0 % |  | Neg | | 0 % | Neg | 0 % |  | | | | |
|  | 7 | **APC Glu1295Glnfs*9** | 17 % | CRT | Neg | 0 % | Neg | 0 % | Surgery | Neg | | 0 % | Neg | 0 % |  | | | | |
|  |  | TP53 Arg306Ter | 10 % |  |  |  | Neg | 0 % |  |  | |  | Neg | 0 % |  | | | | |
|  | 8 | **NRAS Gly13Val** | 13 % | CRT | Pos | 0.8 % | Pos | 0.37 % | Surgery | Neg | | 0 % | Neg | 0 % |  | | | | |
|  |  | TP53 His193Asp | 6 % |  |  |  | Neg | 0 % | Adjuvant therapy |  | |  | Neg | 0 % |  | | | | |
|  | 9 | **TP53 Tyr236Asp** | 55 % | RT | Neg | 0 % | Neg | 0 % | Surgery |  | |  |  |  |  | | | | |
|  |  | **APC Gln1294Glyfs*6** | 13 % |  | Neg | 0 % | Neg | 0 % | Adjuvant therapy |  | |  |  |  |  | | | | |
|  |  | **TP53 Arg175His** | 22 % |  | Pos | 5.4 % | Pos | 1.6 % |  |  | |  |  |  |  | | | | |
|  | 10 | KRAS Ala146Pro | 16 % | RT |  |  | Pos | 2.4 % | Surgery |  | |  |  |  |  | | | | |
|  |  | APC Glu1309Aspfs*4 | 16 % |  |  |  | Pos | 2.5 % |  |  | |  |  |  |  | | | | |
|  | 11 | **TP53 Tyr163Cys** | 27 % | CRT | Pos | 4.1 % | Pos | 1.4 % | Surgery |  | |  |  |  |  | | | | |
|  |  | **TP53 Tyr220Cys** | 59 % |  | Pos | 1.0 % | Pos | 1.7 % | Surgery | Neg | | 0 % | Neg | 0 % |  | | | | |
|  | 12 | **KRAS Gly12Val** | 28 % | RT | Neg | 0 % | Pos | 1.0 % | Adjuvant therapy | Neg | | 0 % | Neg | 0 % |  | | | | |
|  |  | APC Leu1488Tyrfs*19 | 28 % |  |  |  | Pos | 0.2 % |  |  | |  | Neg | 0 % |  | | | | |
|  |  | **TP53 Arg248Trp** | 27 % |  | Neg | 0 % | Neg | 0 % | Surgery |  | |  |  |  |  | | | | |
|  | 13 | APC Ser1355Argfs*18 | 10 % |  |  |  | Neg | 0 % | Adjuvant therapy |  | |  |  |  |  | | | | |
|  |  | FBXW7 Arg465Cys | 12 % |  |  |  | Neg | 0 % |  |  | |  |  |  |  | | | | |
|  |  | **APC Glu1309Aspfs*4** | 70 % |  | Neg | 0 % | Neg | 0 % |  | Neg | | 0 % | Neg | 0 % |  | | | | |
|  | 14 | **JAK2 Val617Phe** | 4 % |  | Pos | 33 % | Pos | 27 % | Surgery | Pos | | 72 % | Pos | 70 % |  | | | | |
|  |  | KRAS Gln61Leu | 44 % |  |  |  | Neg | 0 % |  |  | |  | Neg | 0 % |  | | | | |
|  | 15 | **GNAS Arg201His** | 33 % | CRT | Pos | 8.2 % | Pos | 5 % | Surgery |  | |  |  |  |  | | | | |
|  |  | **KRAS Gly12Val** | 24 % |  | Pos | 9.2 % | Pos | 2.2 % | Adjuvant therapy |  | |  |  |  |  | | | | |
|  |  | **TP53 Pro191del** | 50 % |  | Neg | 0 % | Neg | 0 % |  |  | |  |  |  |  | | | | |
|  | 16 | **NRAS Gly13Asp** | 38 % |  | Neg | 0 % | Pos | 0.42 % | Surgery |  | |  |  |  |  | | | | |
|  |  | APC Arg1114Ter | 33 % |  |  |  | Pos | 0.27 % |  |  | |  |  |  |  | | | | |
|  |  | **NRAS Gly12Asp** | 13 % |  | Pos | 1.0 % | Pos | 0.23 % |  | Pos | | 1.2 % | Pos | 0.29 % |  | | | | |
|  |  | **TP53 Glu204Ter** | 15 % |  | Neg | 0 % | Pos | 0.91 % | Surgery | Pos | | 0.8 % | Neg | 0 % |  | | | | |
|  | 17 | BRAF Asn581Ser | 12 % | CRT |  |  | Neg | 0 % | Adjuvant therapy |  | |  | Neg | 0 % |  | | | | |
|  |  | SMAD4 Ala118Val | 13 % |  |  |  | Pos | 0.47 % |  |  | |  | Pos | 0.56 % |  | | | | |
|  |  | APC Tyr1376Ter | 12 % |  |  |  | Pos | 0.51 % |  |  | |  | Pos | 0.45 % |  | | | | |
|  |  | **PIK3CA Glu545Lys** | 38 % |  | Neg | 0 % | Neg | 0 % |  |  | |  |  |  |  | | | | |
|  | 18 | **KRAS Ala146Thr** | 42 % |  | Neg | 0 % | Neg | 0 % | Surgery |  | |  |  |  |  | | | | |
|  |  | APC Val1320Ter | 35 % |  |  |  | Neg | 0 % |  |  | |  |  |  |  | | | | |
|  |  | FBXW7 Gln277Ter | 31 % |  |  |  | Neg | 0 % |  |  | |  |  |  |  | | | | |
|  |  | **KRAS Gly12Asp** | 12 % |  | Neg | 0 % | Neg | 0 % |  | Neg | | 0 % | Neg | 0 % |  | | | | |
|  | 19 | TP53 Arg337Cys | 21 % | CRT |  |  | Neg | 0 % | Surgery |  | |  | Neg | 0 % |  | | | | |
|  |  | APC Pro1373Leufs*42 | 12 % |  |  |  | Neg | 0 % | Adjuvant therapy |  | |  | Neg | 0 % |  | | | | |
|  |  | FBXW7 Arg479Ter | 17 % |  |  |  | Neg | 0 % |  |  | |  | Neg | 0 % |  | | | | |
|  | 20 | **TP53 Gly245Ser** | 58 % |  | Neg | 0 % | Neg | 0 % | Surgery |  | |  |  |  |  | | | | |
|  |  | **KRAS Gly12Cys** | 41 % |  | Neg | 0 % | Neg | 0 % |  |  | |  |  |  |  | | | | |
|  | 21 | **TP53 Arg273His** | 25 % |  | Neg | 0 % | Neg | 0 % | Surgery+ Adjuv |  | |  |  |  |  | | | | |
|  | 22 | **BRAF Gly466Glu** | 38 % |  | Pos | 0.9 % | Pos | 1 % | Surgery | Neg | | 0 % | Neg | 0 % |  | | | | |
|  |  | TP53 Arg273His | - |  |  |  | Pos | 2 % |  |  | |  | Pos | 4 % |  | | | | |
|  | 23 | **APC Gln1367Ter** | 65 % |  | Neg | 0 % | Neg | 0 % | Surgery |  | |  |  |  |  | | | | |
|  |  | **TP53 Arg282Trp** | 53 % |  | Neg | 0 % | Neg | 0 % |  |  | |  |  |  |  | | | | |
|  | 24 | **KRAS Gly12Asp** | 49 % | RT | Pos | 0.4 % | Neg | 0 % | Surgery |  | |  |  |  |  | | | | |
|  |  | **TP53 Pro152Leu** | 42 % |  | Pos | 0.4 % | Neg | 0 % | Adjuvant therapy |  | |  |  |  |  | | | | |
|  | 25 | **TP53 Arg175His** | 69 % |  | Pos | 0.12 % | Pos | 1 % | Surgery | Neg | | 0 % | Neg | 0 % |  | | | | |
|  |  | APC Leu1488Tyrfs*19 | 25 % |  |  |  | Neg | 0 % |  |  | |  | Neg | 0 % |  | | | | |
|  | 26 | **TP53 Arg248Trp** | 39 % |  | Neg | 0 % | Neg | 0 % | Surgery+ Adjuv |  | |  |  |  |  | | | | |
|  |  | **TP53 Tyr220Cys** | 53 % |  | Pos | 1.2 % | Neg | 0 % | Surgery | Neg | | 0 % | Neg | 0 % |  | | | | |
|  | 27 | PIK3CA His1047Tyr | 28 % |  |  |  | Neg | 0 % | Adjuvant therapy |  | |  | Neg | 0 % |  | | | | |
|  |  | APC Phe1354Ter | 57 % |  |  |  | Neg | 0 % |  |  | |  | Neg | 0 % |  | | | | |
|  |  | **KRAS Gly13Asp** | 20 % |  | Neg | 0 % | Neg | 0 % |  | Neg | | 0 % | Neg | 0 % |  | | | | |
|  |  | TP53 Gly266Val | 23 % |  |  |  | Neg | 0 % |  |  | |  | Neg | 0 % |  | | | | |
|  | 28 | FBXW7 Thr385Ile | 19 % |  |  |  | Neg | 0 % | Surgery |  | |  | Neg | 0 % |  | | | | |
|  |  | APC Thr1438Alafs*39 | 13 % |  |  |  | Neg | 0 % |  |  | |  | Neg | 0 % |  | | | | |
|  |  | GNAS Arg201His | 5 % |  |  |  | Neg | 0 % |  |  | |  | Neg | 0 % |  | | | | |
|  | 29 | **TP53 Arg282Trp** | 41 % |  | Pos | 0.18 % | Neg | 0 % | Surgery | Neg | | 0 % | Neg | 0 % |  | | | | |
|  |  | APC Pro1319Argfs*14 | 29 % |  |  |  | Neg | 0 % |  |  | |  | Neg | 0 % |  | | | | |
|  |  | **APC Gln1303Ter** | 8 % |  | Pos | 0.17 % | Neg | 0 % | Surgery | Neg | | 0 % | Neg | 0 % |  | | | | |
|  | 30 | TP53 Met237Ile | 5 % | CRT |  |  | Neg | 0 % | Adjuvant therapy |  | |  | Neg | 0 % |  | | | | |
|  |  | **KRAS Gly12Val** | 3 % |  | Pos | 0.4 % | Neg | 0 % |  | Neg | | 0 % | Neg | 0 % |  | | | | |
|  | 31 | **BRAF Val600Glu** | 19 % | CRT | Neg | 0 % | Neg | 0 % | Surgery | Neg | | 0 % | Neg | 0 % |  | | | | |
|  |  | **TP53 Arg282Trp** | 18 % |  | Pos | 0.3 % | Neg | 0 % | Adjuvant therapy | Neg | | 0 % | Neg | 0 % |  | | | | |
|  | 32 | FBXW7 Arg465His | 25 % |  |  |  | Neg | 0 % | Surgery |  | |  | Neg | 0 % |  | | | | |
|  |  | **KRAS Gly12Ser** | 25 % |  | Pos | 0.2 % | Neg | 0 % |  | Neg | | 0 % | Neg | 0 % |  | | | | |
|  | 33 | **TP53 Lys132Arg** | 32 % |  | Pos | 0.4 % | Pos | 1 % | Surgery | Neg | | 0 % | Neg | 0 % |  | | | | |
|  |  | APC Glu1309Aspfs*4 | 28 % |  |  |  | Neg | 0 % |  |  | |  | Neg | 0 % |  | | | | |
|  |  | **APC Arg876Ter** | 32 % |  | Pos | 1.5 % | Neg | 0 % | Surgery | Neg | | 0 % | Neg | 0 % |  | | | | |
|  | 34 | APC Gln1367Ter | 31 % |  |  |  | Neg | 0 % | Adjuvant therapy |  | |  | Neg | 0 % |  | | | | |
|  |  | TP53 Thr284Profs*24 | 23 % |  |  |  | Pos | 4.3 % |  |  | |  | Neg | 0 % |  | | | | |
|  |  | TP53 Cys135Ser | 35 % |  |  |  | Neg | 0 % |  |  | |  | Neg | 0 % |  | | | | |
|  | 35 | APC Asn1300Ilefs*5 | 12 % |  |  |  | Neg | 0 % | Surgery |  | |  | Neg | 0 % |  | | | | |
|  |  | **APC Arg876Ter** | 32 % |  | Neg | 0 % | Neg | 0 % |  | Neg | | 0 % | Neg | 0 % |  | | | | |
|  | 36 | **TP53 Arg282Trp** | 64 % |  | Pos | 1 % | Neg | 0 % | Surgery | Neg | | 0 % | Neg | 0 % |  | | | | |
|  | 37 | **TP53 Arg213Leu** | - |  | Neg | 0 % | Neg | 0 % | Surgery | Neg | | 0 % | Neg | 0 % |  | | | | |
|  |  | APC Ser1315Ter | 62 % |  |  |  | Neg | 0 % |  |  | |  | Neg | 0 % |  | | | | |
|  | 38 | **KRAS Gly12Val** | 37 % |  | Neg | 0 % | Neg | 0 % | Surgery | Neg | | 0 % | Neg | 0 % |  | | | | |
|  |  | TP53 Glu294Ter | 31 % |  |  |  | Neg | 0 % |  |  | |  | Neg | 0 % |  | | | | |
|  |  | **TP53 Arg248Gln** | 37 % |  | Neg | 0 % | Neg | 0 % |  | Neg | | 0 % | Neg | 0 % |  | | | | |
|  | 39 | **APC Glu1286Ter** | 34 % |  | Neg | 0 % | Neg | 0 % | Surgery | Neg | | 0 % | Neg | 0 % |  | | | | |
|  |  | TP53 Met133Cysfs*37 | 20 % |  |  |  | Neg | 0 % | Adjuvant therapy |  | |  | Neg | 0 % |  | | | | |
|  |  | **APC Arg876Ter** | 20 % |  | Pos | 1.3 % | Neg | 0 % |  | Neg | | 0 % | Neg | 0 % |  | | | | |
|  | 40 | APC Gln1294Ter | 14 % |  |  |  | Neg | 0 % | Surgery |  | |  | Neg | 0 % |  | | | | |
|  |  | FBXW7 Arg505Cys | 14 % |  |  |  | Neg | 0 % |  |  | |  | Neg | 0 % |  | | | | |
|  | 41 | **TP53 Arg248Gln** | 76 % | CRT | Pos | 0.3 % | Neg | 0 % | Surgery |  | |  |  |  |  | | | | |
|  |  | APC Glu1306Ter | 42 % |  |  |  | Neg | 0 % |  |  | |  |  |  |  | | | | |
|  |  |  |  |  |  |  |  |  |  |  | |  |  |  |  | | | | |

Mutations bolded in blue were used as probes for ctDNA detection with ddPCR.

NT, neoadjuvant therapy. CRT, chemoradiotherapy. RT, radiotherapy. Pos, positive. Neg, negative.

*Information on the allele frequency of one variant in the tumor specimen (#37) and baseline ctDNA with ddPCR (#3) are unavailable for technical reasons. For one patient (#22), one variant (*TP53* Arg273His) was only present in plasma, not in the primary tumor.

VAF, variant allele frequency; ctDNA, circulating tumor DNA; NGS, next generation sequencing; ddPCR, droplet digital PCR.

**Supplementary Table 2** All variants detected in primary tumor tissue and baseline plasmas and their precise variant allele frequencies of the validation group.

|  |  |  |  |  |  |  |
| --- | --- | --- | --- | --- | --- | --- |
|  | **Patient** | **Variant** | **Tumor VAF** | **Baseline ctDNA** | **ctDNA VAF** |  |
|  |  | **KRAS Gly12Val** | 14 % | Negative | 0 % |  |
|  | 1 | APC Thr1493Argfs*14 | 14 % | - | - |  |
|  |  | PIK3CA Glu545Lys | 9 % | - | - |  |
|  |  | **NRAS Gln61Lys** | 18 % | Negative | 0 % |  |
|  | 2 | APC Arg876Ter | 20 % | - | - |  |
|  |  | **TP53 Arg175His** | 20 % | Negative | 0 % |  |
|  | 3 | **TP53 Arg213Ter** | 14 % | Positive | 2.8 % |  |
|  |  | **MET Arg992Thr** | 10 % | Negative | 0 % |  |
|  |  | PTEN Gly132Asp | 15 % | - | - |  |
|  | 4 | PTEN Arg233* | 15 % | - | - |  |
|  |  | **PIK3CA His1047Arg** | 15 % | Positive | 5.9 % |  |
|  |  | **FBXW7 Arg479Gln** | 14 % | Positive | 6.5 % |  |
|  | 5 | **TP53 Arg213Leu** | 22 % | Positive | 1.9 % |  |
|  | 6 | **TP53 Arg110Leufs*13** | 44 % | Positive | 2.7 % |  |
|  |  | **TP53 Arg248Trp** | 41 % | Positive | 1.4 % |  |
|  | 7 | **APC Arg876Ter** | 33 % | Positive | 2.1 % |  |
|  |  | NRAS Gln61Leu | 26 % | - | - |  |
|  |  | PTEN Leu100His | 21 % | - | - |  |
|  | 8 | **FBXW7 Ser582Pro** | 21 % | Positive | 2.3 % |  |
|  |  | **TP53 His193Arg** | 68 % | Positive | 1.2 % |  |
|  | 9 | **TP53 Cys135Tyr** | 24 % | Positive | 5.2 % |  |
|  | 10 | **APC Ser1355Phefs*19** | 27 % | Positive | 0.32 % |  |
|  |  | **FBXW7 Arg479Gln** | 20 % | Positive | 5.3 % |  |
|  | 11 | **NRAS Gly12Asp** | 27 % | Positive | 7.3 % |  |
|  |  | APC Glu1322Ter | 39 % | - | - |  |
|  |  | TP53 Glu349Ter | 39 % | - | - |  |
|  |  | **NRAS Gly12Val** | 35 % | Positive | 0.28 % |  |
|  | 12 | **TP53 Arg273Cys** | 10 % | Negative | 0 % |  |
|  |  | TP53 Glu298Ter | 13 % | - | - |  |
|  | 13 | **KRAS Gly12Val** | 7 % | Negative | 0 % |  |
|  |  | TP53 Arg196Ter | 7 % | - | - |  |
|  |  | APC Gly1357Ter | 42 % | - | - |  |
|  |  | TP53 Arg213Leu | 27 % | - | - |  |
|  | 14 | PTPN11 Tyr80Phe | 23 % | - | - |  |
|  |  | **KRAS Gly12Asp** | 22 % | Positive | 2.4 % |  |
|  |  | FBXW7 Thr402Pro | 17 % | - | - |  |
|  |  | SMAD4 Arg135Ter | 11 % | - | - |  |
|  | 15 | FBXW7 Arg465Cys | 7 % | - | - |  |
|  |  | **KRAS Gly12Ser** | 7 % | Negative | 0 % |  |
|  |  | **KRAS Gly12Ala** | 9 % | Positive | 0.53 % |  |
|  | 16 | **PIK3CA Glu542Lys** | 8 % | Positive | 0.27 % |  |
|  |  | APC Leu1488Tyrfs*19 | 7 % | - | - |  |
|  |  | APC Glu1322Ter | 48 % | - | - |  |
|  | 17 | **TP53 Gly245Ser** | 29 % | Negative | 0 % |  |
|  |  | **NRAS Gln61Lys** | 23 % | Negative | 0 % |  |
|  | 18 | **TP53 Ala129Cysfs*20** | 37 % | Positive | 1.8 % |  |
|  | 19 | **KRAS Gly12Val** | 16 % | Positive | 4.3 % |  |
|  |  | **TP53 Arg175His** | 19 % | Positive | 2.6 % |  |
|  |  | TP53 Arg280Thr | 46 % | - | - |  |
|  | 20 | **APC Glu1309AspfsTer4** | 31 % | Positive | 0.7 % |  |
|  |  | **NRAS Gly12Asp** | 24 % | Positive | 0.4 % |  |
|  | 21 | **TP53 Arg175His** | 24 % | Positive | 1.4 % |  |
|  |  | **APC Glu1309Aspfs*4** | 15 % | Positive | 1.0 % |  |
|  | 22 | **APC Ile1307Ter** | 4 % | Positive | 5.6 % |  |
|  |  | **TP53 Arg175His** | 12 % | Positive | 2.1 % |  |
|  | 23 | **TP53 Tyr220Cys** | 21 % | Positive | 0.24 % |  |
|  |  | **KRAS Gly12Val** | 13 % | Positive | 0.26 % |  |
|  | 24 | **APC Arg876Ter** | 13 % | Positive | 0.34 % |  |
|  |  | **TP53 Cys135Phe** | 21 % | Positive | 1.2 % |  |
|  | 25 | **BRAF Asp594Gly** | 15 % | Positive | 0.67 % |  |
|  |  | FBXW7 Gln388Valfs*4 | 15 % | - | - |  |
|  |  | SMARCB1 Arg377His | 17 % | - | - |  |
|  | 26 | **APC Glu1286Ter** | 13 % | Positive | 0.82 % |  |
|  |  | **KRAS Gly12Ala** | 7 % | Negative | 0 % |  |
|  |  | KRAS Ala146Val | 4 % | - | - |  |
|  |  |  |  |  |  |  |

Variants bolded in blue were used as probes for ctDNA detection.

VAF, variant allele frequency; ctDNA, circulating tumor DNA.
